# Selective killing of ALT-cancer cells by Withaferin-A involves destabilization of Rad51 and disruption of DNA repair

**DOI:** 10.1038/s41419-026-09115-z

**Published:** 2026-07-15

**Authors:** Yue Yu, Huayue Zhang, Jia Wang, Jaspreet K. Dhanjal, Vidhi Malik, Xiupeng Wang, Sunil C. Kaul, Durai Sundar, Renu Wadhwa

**Affiliations:** 1 Molecular Biosystems Research Institute, National Institute of Advanced Industrial Science and Technology (AIST), Tsukuba, Japan; 2 AIST-INDIA DAILAB, Cellular and Molecular Biotechnology Research Institute, AIST, Tsukuba, Japan; 3 Department of Biochemical Engineering & Biotechnology, DAILAB, Indian Institute of Technology (IIT) Delhi, New Delhi, India; 4 Health and Medical Research Institute, AIST, Tsukuba, Japan; 5 Institute of Bioinformatics and Applied Biotechnology (IBAB), Bengaluru, India

**Keywords:** Targeted therapies, Telomeres, Natural products

## Abstract

Alternative lengthening of telomeres (ALT) is a telomerase-independent telomere maintenance mechanism active in a subset of aggressive cancers. Targeting ALT remains a therapeutic challenge due to its complexity and lack of clinically specific inhibitors. Here, we report that the natural compound Withaferin A (Wi-A) selectively impairs ALT cancer cell viability by disrupting Rad51-dependent homologous recombination (HR), a critical pathway for resolving telomeric replication stress and DNA damage in ALT cells. Comparative analysis using isogenic ALT and telomerase-positive cells revealed that Wi-A selectively downregulates PI3K/AKT signaling and induces replication stress, telomeric DNA damage, and DNA repair deficiency in ALT cells. Mechanistically, Wi-A destabilizes the Rad51 nucleoprotein filament and promotes Rad51 degradation via a RFWD3-dependent mechanism localized within ALT-associated PML bodies (APBs). Depletion of either PML or RFWD3 rescues Rad51 stability and reduces sensitivity of ALT cells to Wi-A. Together, these findings uncover a novel mechanism by which Wi-A selectively targets ALT cells through Rad51 destabilization within APBs, highlighting its potential to exploit ALT-specific vulnerabilities in telomere maintenance and DNA repair for therapeutic development.

## Introduction

Telomeres are nucleoprotein complexes that protect chromosome ends from being recognized as DNA double-strand breaks (DSBs). Due to incomplete DNA replication, telomeres progressively shorten with each cell division, eventually triggering DNA damage responses (DDRs) and the consequent induction of cellular senescence or apoptosis [1]. Most cancers bypass this limit by activating telomerase, while around 5–10% utilize a recombination-based mechanism called alternative lengthening of telomeres (ALT) [2]. ALT is predominantly observed in tumors of mesenchymal origin and is associated with poor prognosis in several cancers, including neuroblastoma and certain soft tissue sarcomas [3, 4].

Unlike the well-characterized telomerase-dependent mechanism, the molecular details by which ALT operates remain poorly understood. It is widely accepted that ALT telomere elongation relies on frequent telomeric exchanges between sister chromatids or the copying of telomeric DNA from another chromosome, both of which depend on homologous recombination (HR) [5, 6]. In ALT cells, HR is specifically activated at telomeres and contributes directly to their maintenance [7]. Central to HR is Rad51 recombinase, which forms an ATP-activated filament on single-stranded DNA at sites of damage, facilitating strand invasion and template-guided synthesis for DNA repair [8]. Failure of Rad51 recruitment to telomeres induces elevated telomeric replication stress and DNA damage, which subsequently leads to genomic instability and cell death specifically in ALT cells [9]. Moreover, loss of Rad51 or its paralog Rad51D suppresses DNA repair at telomeres, resulting in telomere shortening and apoptosis[10, 11]. Conversely, Rad51 overexpression enhances HR efficiency, promoting resistance to DNA-damaging therapies [12]. Importantly, Rad51 is broadly expressed in various cancer types [13, 14], with its overexpression linked to low survival rates in cancer patients [15].

Another feature of ALT telomeres is the presence of elevated endogenous telomeric DNA damage, caused by stochastic replication stress [16]. These spontaneous DNA breaks activate DDRs, which subsequently engage homology-directed repair pathways to drive break-induced telomere synthesis. Consequently, ALT telomeres rely on a fragile balance between replication stress and damage-repair mechanisms. Disruption of this equilibrium is central to many strategies proposed for specifically targeting ALT cells [17]. For example, a G-quadruplex-binding cisplatin derivative amplifies telomeric replication stress and suppresses ALT tumor growth in vivo [18]. Similarly, depletion of FANCM, a DNA translocase essential for interstrand crosslink repair, is lethal to ALT cells [19], and antisense oligonucleotides targeting telomere-damage-induced long non-coding RNAs provoke ALT-specific cell death by further elevating replication stress [9]. In addition to these, other strategies have been proposed to exploit ALT dependencies, including TSPYL5 depletion to trigger POT1 degradation [20], inhibition of the p53-AKT pathway [21], and targeting the exonuclease EDX2 [22]. However, despite these promising leads, none have progressed to clinical application, highlighting an urgent need for novel molecular targets and therapeutics against ALT-driven cancers.

Withaferin-A (Wi-A) is the major bioactive withanolide derived from Ashwagandha (*Withania somnifera*), a medicinal herb traditionally used in India Ayuvedic system. Wi-A has demonstrated significant pharmacological benefits, particularly its potential as a therapeutic agent in cancer treatment. Research across various cancer models has highlighted its diverse activities in DDR, including the induction of reactive oxygen species (ROS) [23], modulation of key DDR proteins [24, 25], and strong synergistic effects with radiation or DNA-crosslinking agents [26, 27]. These effects, however, are not specific to ALT cells. To uncover therapeutic vulnerabilities of ALT cancers to Wi-A, we previously conducted a study using isogenic telomerase-plus (TEP) and -minus (ALT) cells and reported that Wi-A exhibited higher toxicity to ALT cells [28]. In the present study, we investigated the impact of Wi-A on DNA repair mechanisms. We demonstrate that Wi-A causes Rad51-dependent HR deficiency, which leads to DNA replication stress preferentially in ALT cells and persistent DNA damage at ALT telomeres. Mechanistically, we provide evidence that Wi-A facilitates the interaction between Rad51 and the E3 ligase RFWD3 in ALT-associated PML bodies, resulting in enhanced Rad51 degradation exclusively in ALT cells.

## Results

### Wi-A causes dysregulation of PI3K-Akt pathway in ALT cells

**Fig. 1:**
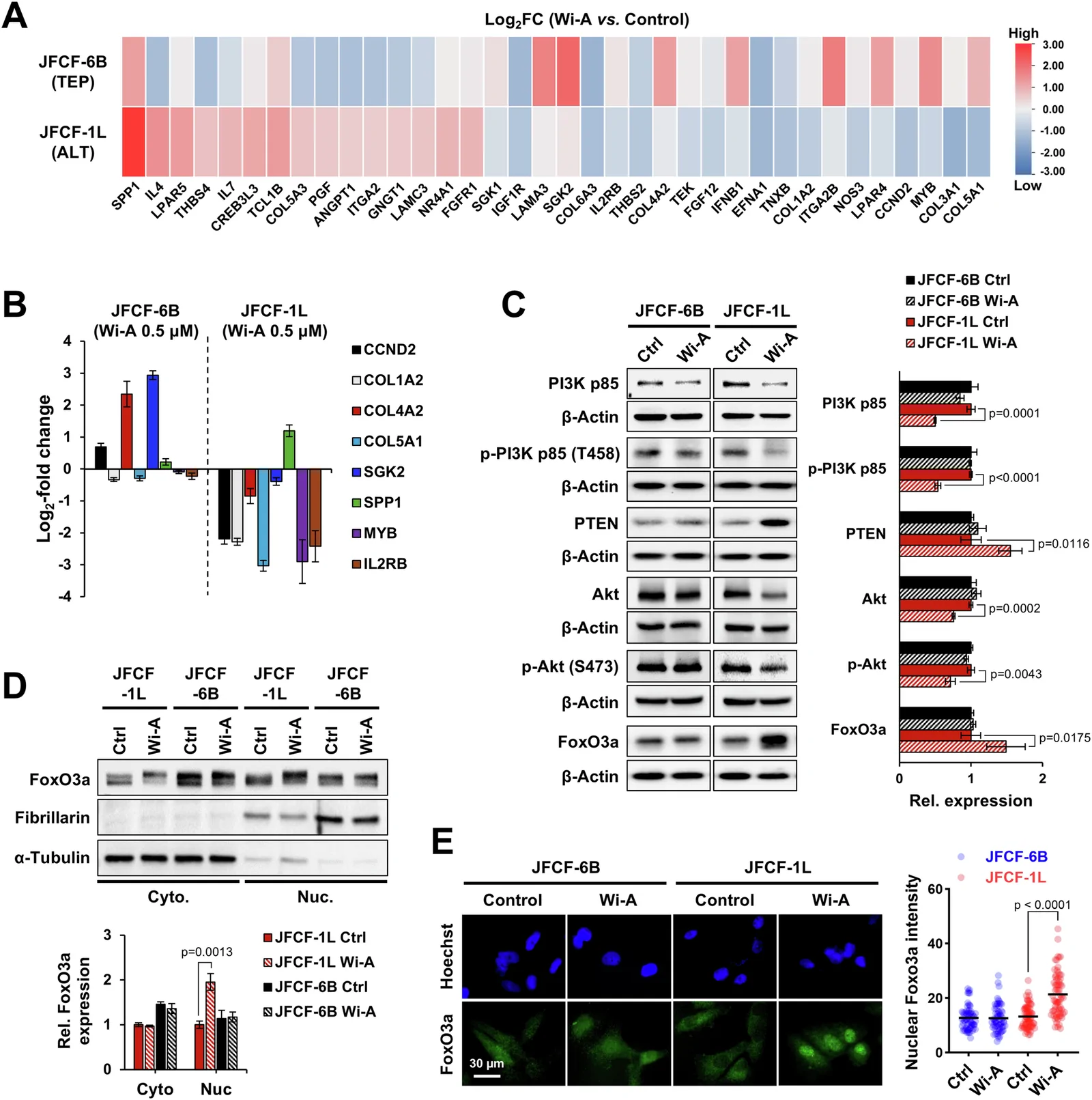
Wi-A causes dysregulation of PI3K-Akt pathway in ALT cells. **A** Comparison between control and Wi-A-treated cells showing stronger inhibition of PI3K pathway in ALT cells upon Wi-A treatment (0.5 μM, 24 h). The fold change values were log2 transformed, and heatmap was prepared. **B** RT-qPCR validation of microarray data showing differential transcript levels of PI3K pathway related genes in Wi-A treated (0.5 μM, 24 h) TEP and ALT cells (mean ± s.d., *n* = 3 independent experiments). **C** Western blot analysis for proteins related to PI3K-Akt pathway showing downregulation in the expressions of total and phosphorylated PI3K and Akt, and upregulations in PTEN and Foxo3a, in Wi-A treated (0.5 μM, 24 h) ALT cells. Their expression remained largely unchanged in TEP cells. Quantitation from three independent experiments is shown on the right (mean ± s.d., normalized to control) and compared using Student’s two-tailed *t*-test. **D** Western blot analysis showing an increase in nuclear levels of Foxo3a in ALT, but not TEP, cells treated with Wi-A (0.5 μM) for 24 h. Fibrillarin and α-Tubulin were used as loading controls for nuclear and cytoplasm protein fractions, respectively. Quantitation from three independent experiments is shown below (mean ± s.d.), and compared using Student’s two-tailed *t*-test. **E** Immunostaining showing nuclear accumulation of Foxo3a in Wi-A treated ALT cells only. TEP and ALT cells were treated with equivalent dose of Wi-A (0.5 μM) for 24 h. Quantification of at least 50 randomly selected nuclei per treatment is shown on the right (one-way ANOVA test).

We previously showed that SV40-immortalized ALT cells (JFCF-1L) are more sensitive to Wi-A-induced cytotoxicity than their isogenic TEP counterparts (JFCF-6B) [28]. To explore the molecular mechanism(s) of this ALT cell-selective toxicity, we performed cDNA array analysis of Wi-A (0.5 μM, selectively toxic to ALT cells)-treated TEP and ALT cells. Soft clustering analysis with Mfuzz categorized all genes into nine expression clusters (Fig. S1A); clusters 4 (1907 genes) and 9 (2661 genes), which showed marked changes in ALT but not TEP cells, were subjected to Kyoto Encyclopedia of Genes and Genomes (KEGG) enrichment analysis. The result revealed 96 and 82 significantly enriched pathways (corrected *P* < 0.05) for cluster 4 and 9, respectively, with the top 30 shown in Fig. S1B, C. Notably, 39 genes within the PI3K-AKT pathway were downregulated selectively in ALT cells. Given the oncogenic role of PI3K-AKT signaling [29], we examined this pathway in greater depth. As shown in Fig. 1A, an abreast comparison of significantly altered PI3K-AKT pathway genes (log2-fold change ≥ 1) following Wi-A treatment suggested a profound differential effect on this pathway in ALT versus TEP cells. We validated eight genes showing more than twofold differences by qRT-PCR. As shown in Fig. 1B, four genes (COL1A2, COL5A1, MYB, IL2RB) were more strongly downregulated and one (SPP1) was more strongly upregulated in ALT cells. Three others (CCND2, COL4A2, SGK2) showed opposite regulation between the two cell types (Fig. 1B). These results endorsed the microarray data, confirming differential regulation of PI3K-AKT signaling in Wi-A-treated TEP and ALT cells.

Based on the above findings that Wi-A causes stronger dysregulation on PI3K-associated genes in ALT cells, we next examined protein levels of PI3K and its major downstream effectors. Western blotting revealed decreased PI3K p85 and its phosphorylation at Tyr458 in Wi-A-treated ALT cells, accompanied by a sharp induction of PTEN, a negative regulator of PI3K (Fig. 1C). Total and phosphorylated Akt (Ser473) of ALT cells were also downregulated in response to Wi-A treatment. In contrast, TEP cells showed no noticeable difference in the expression of these proteins (Fig. 1C). FoxO3a, a key downstream target of the PI3K-Akt pathway, whose cytoplasmic sequestration and degradation are frequent in cancers [30], was increased in Wi-A-treated ALT but not TEP cells (Fig. 1C). This upregulation coincided with stronger nuclear accumulation in ALT cells (Fig. 1D, E). Together, these results indicate that Wi-A preferentially suppresses PI3K-Akt signaling in ALT cells.

### Wi-A provokes replication stress-associated DNA breaks in ALT cells

**Fig. 2:**
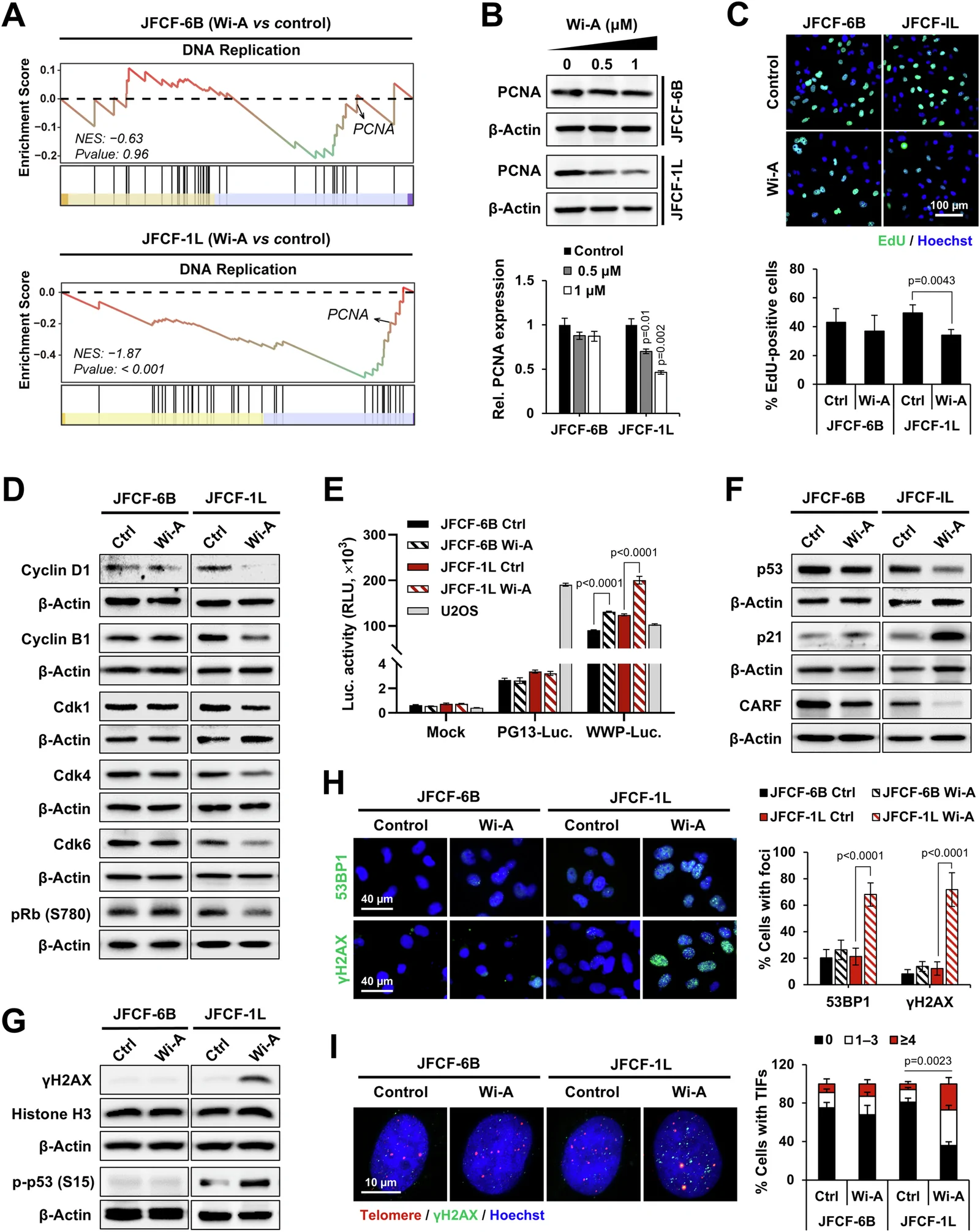
Wi-A triggers replication stress-mediated DNA damage in ALT cells. **A** GSEA of transcriptional profiles using KEGG gene sets showing insignificant regulation of DNA replication–associated genes in TEP cells (upper) but significant inhibition in ALT cells (lower) following Wi-A treatment (0.5 μM, 24 h). PCNA is highlighted as a representative gene. **B** Western blotting showed Wi-A treatment reduced PCNA protein levels selectively in JFCF-1L ALT cells. Quantitation was performed using one-way ANOVA test against the relevant controls (mean ± s.d.; *n* = 3 independent experiments). **C** EdU staining of control and Wi-A-treated TEP and ALT cells showed decreased number of EdU-positive proliferating cells in ALT cells following Wi-A treatment (0.5 μM, 24 h). Quantitation is shown below (mean ± s.d., *n* = 3 independent experiments, Student’s two-tailed *t*-tests). **D** Western blotting of proliferation marker proteins in control and Wi-A-treated (0.5 μM, 24 h) TEP and ALT cells showing decrease in their expressions in ALT, but not TEP, cells. **E** Wild type p53-dependent luciferase reporter assays showed no change in the PG13-driven luciferase activity, but activation in the WWP-driven luciferase in Wi-A-treated (0.5 μM, 24 h) ALT cells. Data quantified from 3 independent experiments are shown as mean ± s.d.(one-way ANOVA test). U2OS cells with wild type p53 were used as positive control. **F** Western blotting showed reduction in mutant p53 in Wi-A-treated (0.5 μM, 24 h) ALT cells only. A higher induction of p21 and stronger repression of CARF by Wi-A was observed in ALT cells, as compared to their TEP counterparts. Since p53 and Cdk1 (**D**) were detected on the same blot, β-actin is same. **G** Western blot showing elevated γH2AX and phospho-p53 (Ser15) levels in ALT cells following 24 h treatment with 0.5 μM Wi-A. **H** Immunostaining of 53BP1 and γH2AX showing increase in their focus formation after Wi-A treatment (0.5 μM, 24 h) in ALT cells only. Number of 53BP1 and γH2AX foci positive cells were quantified (mean ± s.d., Student’s two-tailed *t*-test), wherein at least 100 nuclei cells were scored per sample. **I** Co-immunostaining of telomeres and γH2AX showing Wi-A-induced (0.5 μM, 24 h) telomere damage in ALT cells. Co-localizing signals were quantified as telomere dysfunction-induced foci (TIFs) (mean ± s.d.), with at least 100 nuclei analyzed per sample. The proportion of cells with ≥4 TIFs was compared with control using a Student’s two-tailed *t*-test. Telomeres were labeled using antibodies against TRF1 and TRF2.

PI3K-Akt pathway is crucial for maintaining genomic stability through its control of multiple cell-cycle processes, including DNA replication [29]. Consistent with this, Gene Set Enrichment Analysis (GSEA) on KEGG gene sets revealed that Wi-A significantly inhibited DNA replication in ALT but not TEP cells (Fig. 2A). This was supported by a dose-dependent reduction in PCNA (a central DNA replication factor) and by impaired DNA synthesis specifically in Wi-A-treated ALT cells (Fig. 2B, C). In parallel, Wi-A caused remarkably higher impairment of proliferation in ALT, associated with downregulation of cyclins and Cdks, as well as their downstream factor pRb (Ser780), leading to selective growth arrest (Figs. 2D and S2A–C).

Our earlier studies have shown (i) mutant status of p53 in JFCF-6B (TEP cells) and JFCF-1L (ALT cells) [28] and (ii) restoration of wild-type p53 activity by Wi-A in cancer cells harboring a p53^Y220C^ mutation [24]. We therefore reasoned that stronger growth arrest in Wi-A-treated ALT cells may be mediated by restoration of wild-type p53 function. However, as shown in Fig. 2E, reporter assays using PG13-Luc (wild-type p53 consensus) showed no change in luciferase activity in either cell type upon Wi-A treatment. On the other hand, WWP-Luc (p21 promoter) was activated by Wi-A and showed stronger activation in ALT as compared to TEP cells, suggesting p53-independent induction of p21 (Fig. 2E). Western blotting indeed appraised stronger upregulation of p21 in ALT cells (Figs. 2F and S2D). We further examined CARF that has been shown to act as transcriptional repressor of p21 [31], and found a markedly stronger reduction in CARF expression in Wi-A-treated ALT cells than in TEP cells (Figs. 2F and S2D). These data suggested that the replication stress-induced growth arrest in Wi-A-treated ALT cells is mediated through activation of CARF-p21-Cyclin/CDK-Rb axis.

Given the role of CARF in DNA damage signaling [32] and the fact that replication stress can drive genomic instability, we next asked whether Wi-A elicits a DDR in TEP and ALT cells. Western blotting revealed a marked increase in γH2AX specifically in ALT cells after Wi-A treatment (Figs. 2G and S2E). Of note, ALT cells also showed enhanced p53 Ser15 phosphorylation, consistent with its function as a DSB marker independent of p53 mutation status [33]. To further assess DNA damage, we quantified 53BP1 and γH2AX nuclear foci. Wi-A caused a pronounced increase in foci-positive ALT cells (21% to 68% for 53BP1; 12% to 71% for γH2AX), whereas TEP cells showed minimal response (Fig. 2H). To determine whether this susceptibility extends to telomeres, we examined the effect of Wi-A on telomere integrity using telomere-dysfunction-induced foci (TIF) analysis. As shown in Fig. 2I, Wi-A had no detectable effect on TEP cells but significantly increased the fraction of TIF-positive ALT cells. Collectively, these data suggested that Wi-A, within a 24 h treatment period, induced both global and telomeric DNA damage in ALT, but not TEP, cells.

### Wi-A impedes repair in ALT cells by inhibiting HR

**Fig. 3:**
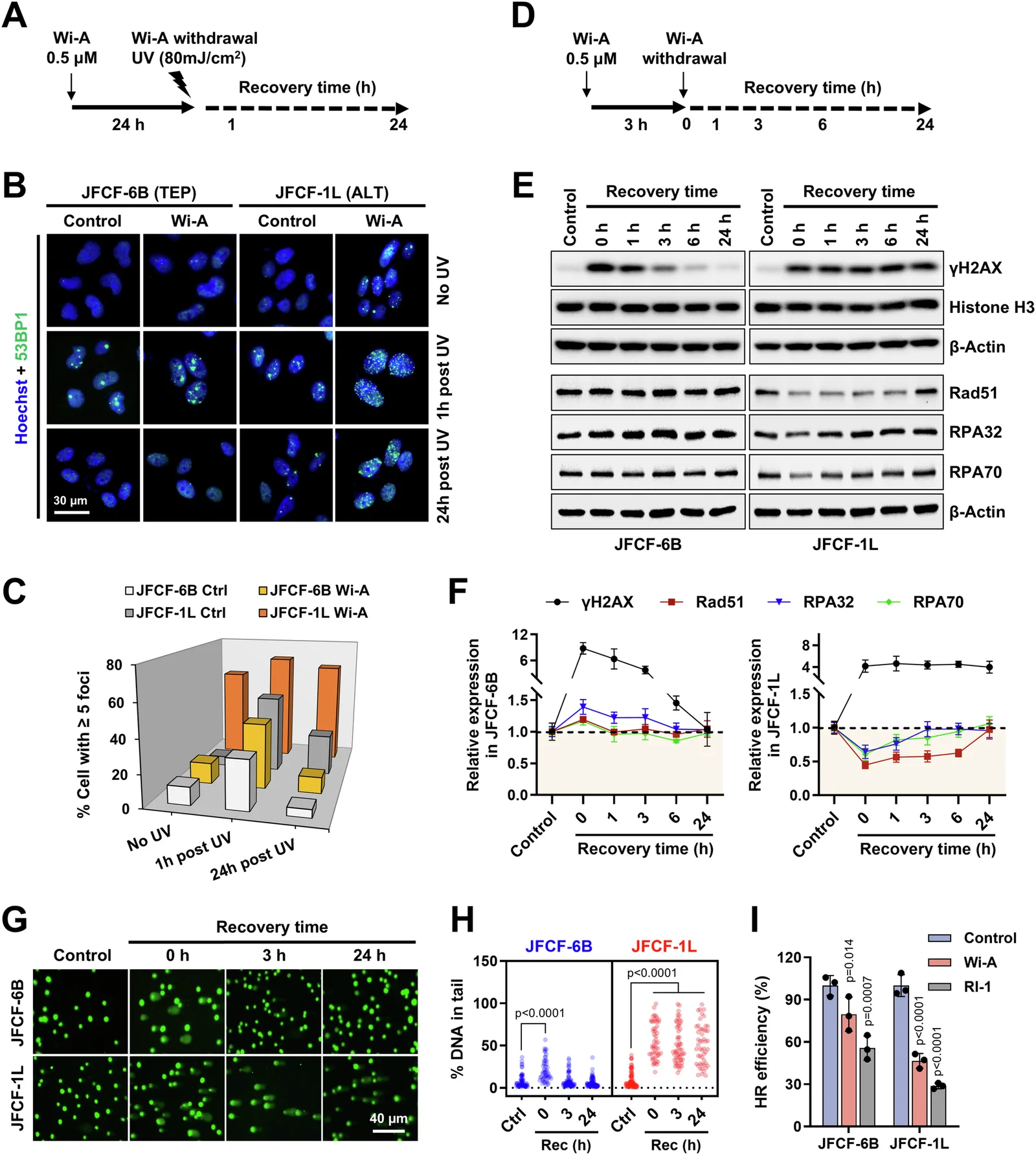
Wi-A impairs DNA repair in ALT cells by inhibiting homologous recombination. **A** Experimental scheme for induction and repair of 53BP1 foci. Cells were treated with Wi-A at 0.5 μM for 24 h, followed by UV exposure at a dose of 80 mJ/cm^2^. Cells were subjected to immunostaining after 1 and 24 h recovery in drug-free medium. **B** Immunostaining of 53BP1 showing UV-induced foci nearly disappeared after 24 h recovery in Wi-A treated TEP cells but was sustained in Wi-A treated ALT cells. **C** Quantitation of cells with at least 5 of 53PB1 foci is shown. A total of at least 100 nuclei were analyzed for each sample. **D** Schematic of experiment plan. Cells were exposed to Wi-A pulse treatment for 3 h at 0.5 μM and harvested at indicated time points after drug withdrawal. **E** Western blot analysis of kinetic alterations in expression levels of proteins involved in HR pathway. The data indicate that ALT cells after release from Wi-A treatment showed persistent induction of γH2AX, along with delayed restoration of Rad51, PPA32 and RPA70. **F** Quantitation of their expression levels in TEP (left panel) and ALT (right panel) cells is shown (mean ± s.d., *n* = 3 independent experiments). γH2AX was normalized to Histone H3. **G** Kinetics of DSBs measurement by comet assay showing impaired DNA repair capacity in Wi-A-treated JFCF-1L ALT cells only. Cells were treated with Wi-A at 0.5 μM for 3 h, and allowed for recovery in drug-free medium for the indicated time. **H** Quantitation of at least 60 cells from three independent experiments is shown and compared by one-way ANOVA test. **I** Efficiency of HR-mediated DNA repair in control and Wi-A-treated TEP and ALT cells showing stronger decrease in the latter. Cells carrying DR-GFP reporter were transfected with DSB-inducible I-SceI plasmid and on the following day were treated with Wi-A at 0.25 μM for 24 h. The repair efficiency was scored as the percentage of fluorescence intensity of GFP in control cells (mean ± s.d., one-way ANOVA test to control). An established HR inhibitor RI-1(5 μM for 24 h) was used as a positive control.

ALT cells have been shown to extend telomeres particularly through exploitation of DNA repair machinery [17]. We next considered whether Wi-A affected DNA repair differentially in TEP and ALT cells. Because UV irradiation induces DNA damage that engages multiple repair pathways [34], we used it to compare the global repair capacity between the two cell types. Accordingly, cells were pretreated with Wi-A for 24 h, followed by UV exposure, and repair progression was monitored by 53BP1 foci either immediately or after 24 h recovery in drug-free medium (Fig. 3A). In TEP cells, UV-stimulated 53BP1 foci almost disappeared after 24 h recovery, regardless of Wi-A pretreatment (Fig. 3B, C), indicating little effect of Wi-A on their repair capacity. On the other hand, in ALT cells, high foci levels were induced at 1 h post UV irradiation. At 24 h, the control ALT cells repaired most of the DNA breaks, whereas Wi-A-treated ALT cells showed substantial retention of 53BP1 foci, implicating persisted DNA damage (Fig. 3B, C). Similar results were observed following exposure to ionizing radiation (IR), a potent inducer of DSBs. As shown in Fig. S3, TEP cells showed a marked reduction in DNA damage under both control and Wi-A-treated conditions, as indicated by decreased γH2AX levels at 24 h post IR (Fig. S3B). In contrast, Wi-A-treated ALT cells retained elevated γH2AX levels (Fig. S3C), indicating a selective defect in DNA damage resolution. These results suggested that Wi-A caused ineffective repair of DNA damage in ALT, but not TEP, cells.

HR, as one of the DNA repair pathways, is an established critical factor for maintaining ALT telomeres [11]. Based on the above data, we reasoned that Wi-A might weaken HR activity, thereby inducing a stronger influence on ALT, as compared to TEP cells. To test this, we induced repairable DSBs by short Wi-A treatment (3 h; 0.5 μM) followed by recovery in 24 h, and examined HR proteins in TEP and ALT cells (Fig. 3D). As expected, in TEP cells, Wi-A induced phosphorylation of H2AX was readily detected upon treatment and gradually decreased during recovery duration reaching control levels in 24 h (Fig. 3E, F). In contrast, Wi-A pulse treatment (3 h) in ALT cells led to sustained induction of γH2AX, at least for a period of 24 h after drug removal, and concomitantly showed reduction in the expression of proteins involved in HR. Of note, Rad51 showed the strongest decrease (Fig. 3E, F). Furthermore, comet assays performed under the same conditions revealed that TEP cells began repairing Wi-A-induced damage within 3 h of drug withdrawal, whereas ALT cells retained substantial DSBs even after 24 h (Fig. 3G, H), indicating a severe repair defect induced by Wi-A in these cells. To directly quantify HR activity, we employed a plasmid-based repair reporter assay [35]. As shown in Fig. 3I, the HR efficiency observed in ALT cells was significantly reduced following Wi-A treatment, comparable to the inhibition achieved with the known HR inhibitor RI-1. Only a moderate decrease was found in TEP cells after the same treatment (Fig. 3I). Altogether, the above data suggested that Wi-A-induced defects in DNA repair of ALT cells is mediated by HR inhibition.

### Wi-A attenuates Rad51 activity, inducing DNA damage hypersensitivity in ALT cells

HR relies on Rad51 nucleofilament formation on single-stranded DNA, driven by Rad51 subunit interactions and recruitment to DNA damage sites by BRCA2 [36]. To investigate whether Wi-A disrupts the Rad51-BRCA2 complex, molecular docking analysis was performed. The results showed that Wi-A interacted with four critical residues (Phe1524, Ala1527, Leu1545, and Phe1546), forming three main points of contact between BRCA2-Rad51 proteins, with the binding energy of −2.88 Kcal/mol. Superimposition of the Rad51-Wi-A-BRCA2 complex confirmed stable Wi-A binding at the Rad51-BRCA2 interface (Fig. S4A). However, the binding energy between BRCA2 and Rad51 was only slightly reduced (from −10.4 Kcal/mol to −10.2 Kcal/mol) in the presence of Wi-A. Therefore, we concluded that Wi-A does not disrupt BRCA2-Rad51 interactions.

**Fig. 4:**
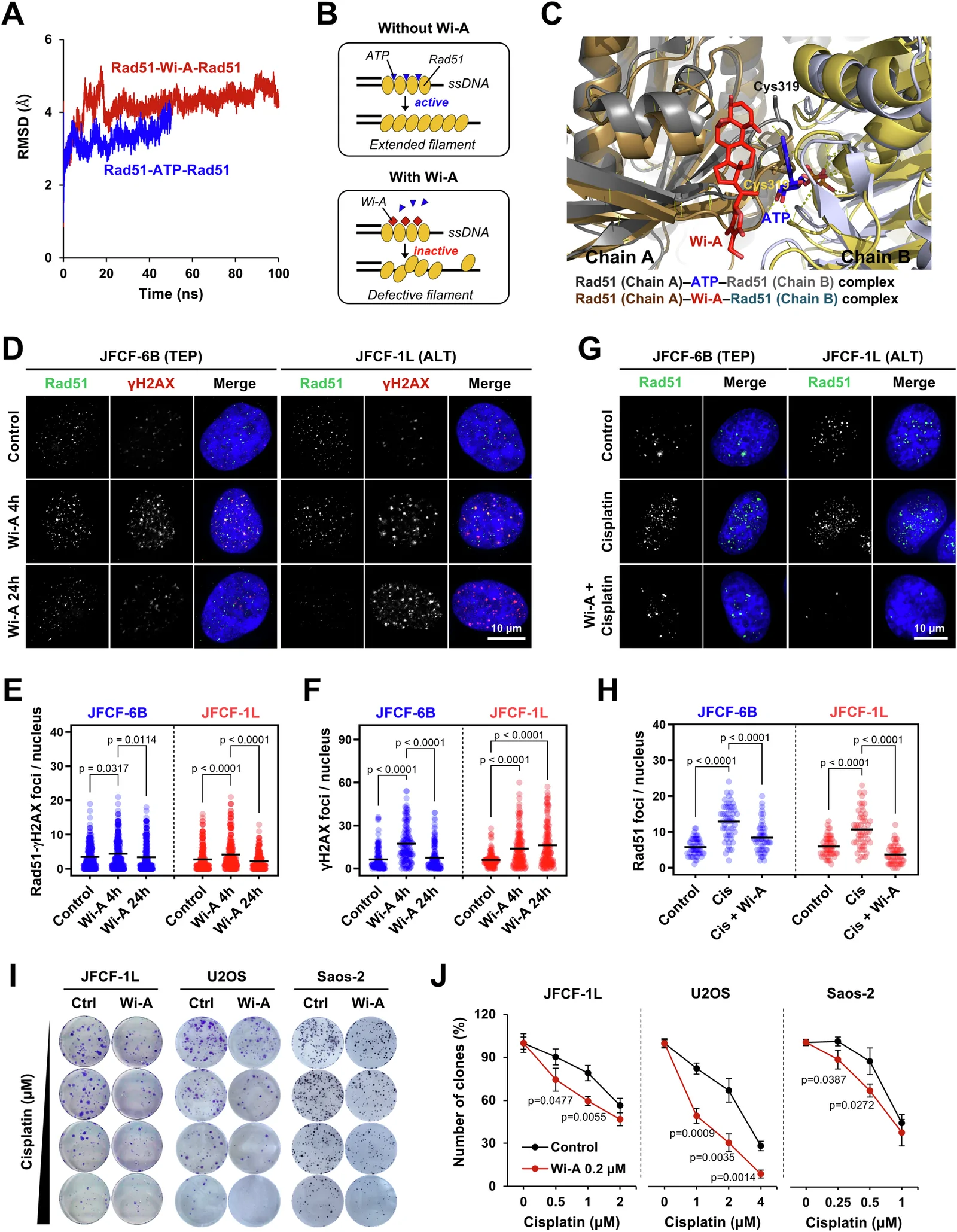
Wi-A attenuates Rad51 activity, rendering ALT cells hypersensitive to DNA crosslinking agents. **A** RMSD plot of Rad51-ATP-Rad51 complex (blue) and Rad51-Wi-A-Rad51 complex (red) for simulation trajectory of 50 ns and 100 ns respectively. **B** Schematic model showing impact of Wi-A on Rad51 filament formation: Wi-A binding at Rad51 protomer-protomer interface instead of ATP leads to aberrant Rad51 filament structures. **C** Superimposed image of Rad51-ATP-Rad51 complex (dark grey-blue-light grey) with that of Rad51-Wi-A-Rad51 complex (brown-red-yellow). **D** Immunostaining of Rad51 and γH2AX showing rapid (within 4 h) colocalization in 0.5 μM Wi-A-treated TEP and ALT cells, which dissipated after 24 h, leaving persistent γH2AX foci in ALT cells only. Quantification of Rad51-γH2AX colocalized foci (**E**) and γH2AX foci (**F**) are shown (mean ± s.d.) and compared by one-way ANOVA, with at least 200 nuclei scored per sample. **G** Immunostaining of Rad51 showing that Wi-A disrupts assembly of DSB-induced Rad51 foci. Cells were sequentially treated with 10 μM cisplatin for 6 h, followed by 0.5 μM Wi-A for 24 h. **H** A total of at least 50 nuclei per treatment were randomly selected and quantified below (one-way ANOVA test). **I** Colony forming assay showing Wi-A sensitizes ALT cell lines (JFCF-1L, U2OS and Saos-2) to DNA cross-linking agent cisplatin. Cells were incubated for 6 h in media containing 0.2 μM Wi-A and/or varying concentrations of cisplatin, and were then allowed to grow in drug-free media for an additional 7–10 days. **J** Average number of clones for each condition is quantified and normalized to the 0 μM cisplatin of that condition (mean ± s.d., *n* = 3 independent experiments, unpaired *t* test to control).

The nucleotide state at the Rad51 protomer-protomer interface determines the conformation of Rad51 nucleoprotein filaments: ATP binding supports the active, extended filament, whereas ADP favors the inactive, condensed state [37]. To test whether Wi-A disrupts Rad51 function through ATP depletion, we measured intracellular ATP levels in control and Wi-A-treated TEP and ALT cells, but detected no significant changes in either cell type (Fig. S4B). We next examined whether Wi-A interferes directly with Rad51-Rad51 interactions by targeting the ATP-binding site at the protomer interface. Docking analysis revealed that Wi-A was stably interacting with this site with a high binding affinity of -41.02 Kcal/mol by hydrophobically interacting with the majority of ATP-interacting residues of Rad51 (Fig. 4A and Table S1). Furthermore, Wi-A increased the Rad51-Rad51 binding energy (−17.7 kcal/mol) relative to ATP (−15.8 kcal/mol) (Table S1), suggesting that Wi-A, when occupying the ATP-binding site, may favor formation of an inactive Rad51 filament (Fig. 4B), consistent with reports that defective ATP binding destabilizes Rad51 filaments [38]. Of note, Wi-A also interacted with the Cys319 residue at the interface of Rad51 protomers (Fig. 4C), just like inhibitor RI-1, which covalently binds to this reside and destabilizes nucleofilament through disrupting Rad51-Rad51 interaction [39].

Rad51 recruitment to and retention at DSBs is essential for its recombinase activity during DNA repair. To determine whether Wi-A impairs Rad51 function in TEP and ALT cells by destabilizing Rad51 filaments, we performed immunofluorescence microscopy to monitor the dynamics of Rad51 foci formation. Microscopy images and quantification showed rapid recruitment of Rad51 to DNA breaks after 4 h of Wi-A treatment in both cell types (Fig. 4D, E). By 24 h, however, Rad51 foci in ALT cells became strikingly reduced compared to 4 h, despite persistent DNA damage (Fig. 4D, F), indicating Wi-A-mediated Rad51 filament disassembly and consequent loss of Rad51 function. TEP cells also showed reduced Rad51 foci at 24 h, but likely due to the recovery from Wi-A-induced damage (Figs. 4D, F and S4C). To further validate this effect, we induced universal DNA damage using cisplatin and examined Rad51 foci formation in the presence or absence of Wi-A. Co-treatment with Wi-A markedly reduced cisplatin-induced Rad51 foci in both TEP and ALT cells (Fig. 4G, H), confirming Wi-A-mediated inhibition of Rad51 filament stability. Notably, ALT cells showed a more pronounced reduction after the combined treatment, with Rad51 foci becoming nearly undetectable (Fig. 4G).

Abrogation of Rad51 function can lead to cells' hypersensitivity to DNA crosslinking agents [39, 40]. We hence hypothesized that Wi-A-induced Rad51 disruption in ALT cells may potentiate the lethal effects of cisplatin. To examine if Wi-A works in this modality, we performed a colony-forming assay using three ALT cell lines (JFCF-1L, U2OS and Saos-2) following Wi-A treatment alone or in combination with cisplatin. As expected, Wi-A significantly increased the sensitivity of all three cell types to cisplatin (Fig. 4I, J).

Taken together with the results from molecular docking and biochemical analysis, these data led us to conclude that Wi-A impaired Rad51 activity, with a stronger impact on ALT cells, thereby rendering them more susceptible to the drugs targeting DNA interstrand crosslinks.

### Wi-A promotes PML-dependent destabilization of Rad51 in ALT cells

**Fig. 5:**
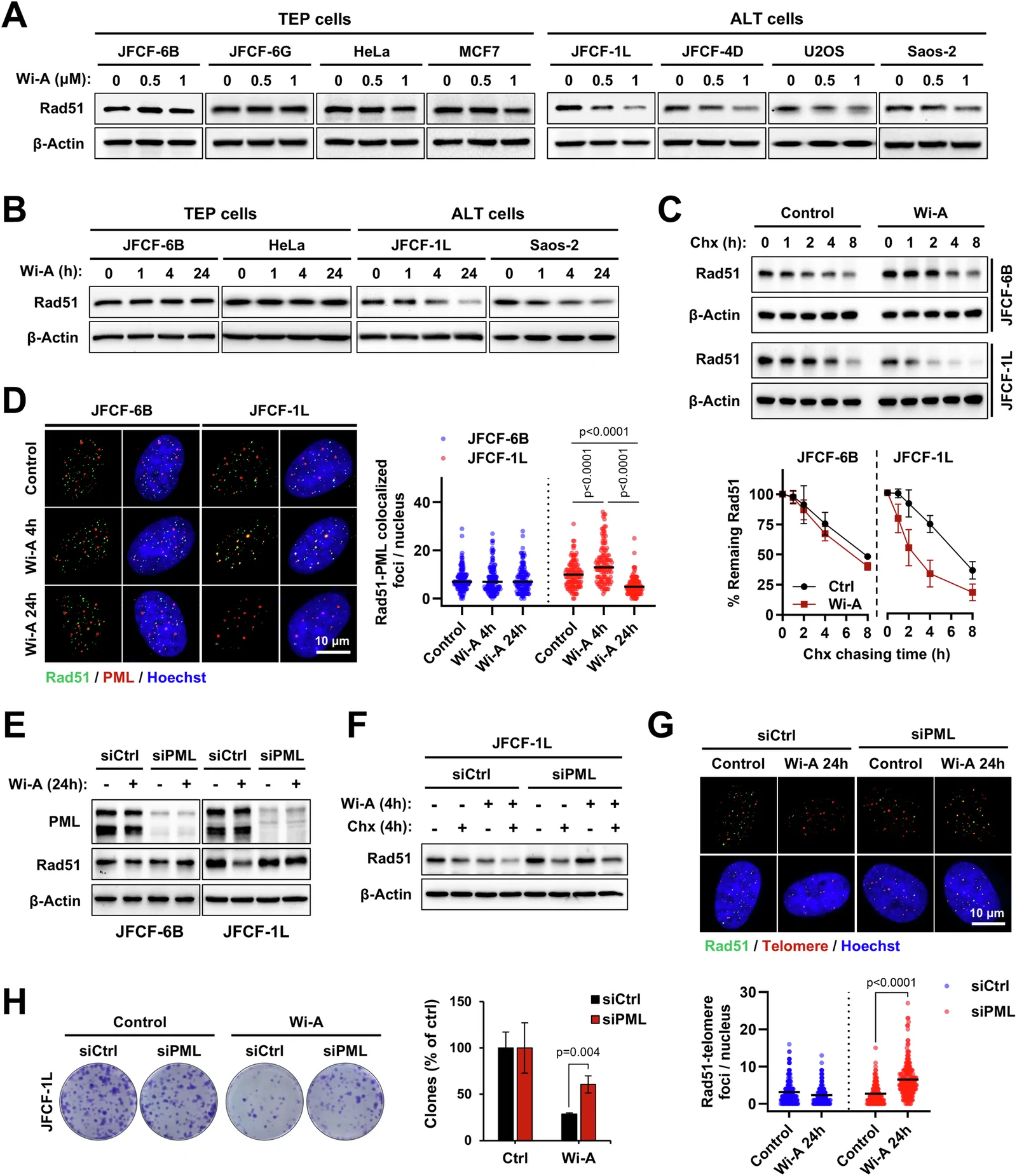
Wi-A induces PML-dependent Rad51 degradation in ALT cells. **A** Wi-A downregulates Rad51 expression in a dose-dependent manner in ALT, but not TEP, cells. Cells were treated with the indicated concentrations of Wi-A for 24 h and harvested for Western blot analysis. Since Rad51 and PCNA (Fig. 2D) were detected on the same blot for JFCF-6B and JFCF-1L, β-actin serves as the shared loading control. **B** Western blot analysis of TEP and ALT cells showing a time-dependent reduction in Rad51 expression in ALT cells following Wi-A treatment (0.5 μM). **C** Wi-A promotes Rad51 degradation in JFCF-1L ALT cells. Control and Wi-A (0.5 μM) treated cells were chased up to 8 h in the presence of 10 μg/mL cycloheximide (CHX) and analyzed by western blotting. The ratio of Rad51 to β-actin band intensity was normalized to the starting point of the chase. Relative changes over time were quantified (mean ± s.d., *n* = 3 independent experiments) and are shown below. **D** Immunofluorescence of Rad51 and PML showing increased colocalization after 4 h of Wi-A treatment in ALT cells but disappearing with extended treatment up to 24 h. Quantification of at least 100 randomly selected nuclei per treatment is shown on the right (one-way ANOVA test). **E** PML knockdown rescues Wi-A-mediated Rad51 inhibition in ALT cells. Cells were transiently transfected with siRNAs for 24 h, followed by treatment with 0.5 μM Wi-A for an additional 24 h. Cell lysates were immunoblotted with indicated antibodies. **F** PML knockdown in ALT cells mitigates Wi-A-induced Rad51 degradation. JFCF-1L cells were transfected with control or PML siRNAs, treated with 0.5 μM Wi-A, 10 μg/mL CHX, or their combination, and analyzed by Western blotting with the indicated antibodies. **G** Immunofluorescence showing Rad51 recruitment to ALT telomeres in siPML‑transfected JFCF‑1L cells after Wi‑A treatment (0.5 μM, 24 h). Quantification of ≥200 randomly selected nuclei per treatment is shown below (one‑way ANOVA test). **H** Colony forming assay showing reduced toxicity of Wi-A in JFCF-1L cells transfected with PML siRNA. siRNA-transfected cells were treated with 0.2 μM Wi-A for 4 h, then cultured in drug-free media. Colonies formed after 10 days were quantified (mean ± s.d., *n* = 3 independent experiments, Student’s two-tailed *t*-test).

To elucidate the Rad51-related mechanism underlying the differential sensitivity of TEP and ALT cells to Wi-A, we assessed Rad51 expression following Wi-A treatment in a panel of TEP and ALT cell lines. As shown in Figs. 5A and S5A, Rad51 levels remained largely unaffected in TEP cells, while a dose-dependent reduction was observed in ALT cells. Interestingly, this reduction in Rad51 occurred rapidly, within 4 h of Wi-A treatment (Figs. 5B and S5B), whereas Rad51 mRNA levels were unchanged under the same conditions (Fig. S5C), suggesting Wi-A may inhibit Rad51 of ALT cells through a transcription-independent mechanism. To investigate further, we performed a cycloheximide (CHX) chase assay to evaluate Rad51 stability. During the 8 h chase period, the half-life of Rad51 protein was significantly reduced by Wi-A treatment in ALT cells (from 7 h to 3 h), but not in TEP cells (Fig. 5C). Moreover, MG132, a potent proteasome inhibitor, effectively blocked Wi-A-mediated Rad51 degradation in ALT cells (Fig. S5D). These results demonstrate that Wi-A promotes proteasomal degradation of Rad51 during the early phase of the treatment in ALT cells.

Next, we sought to understand why Rad51 degradation, induced by Wi-A treatment, occurs exclusively in ALT cells. ALT telomeres are uniquely associated with PML bodies, which are enriched with ubiquitinating enzymes and exhibit proteasomal activity [41, 42]. Proteins bound at ALT telomeres are more prone to proteasomal degradation [20, 43]. We found that, in Wi-A-treated ALT cells, Rad51 rapidly (within 4 h) accumulated at damaged telomeres (Figs. 4D and S5E, F), suggesting its recruitment to PML bodies and susceptibility to proteasomal degradation. Consistently, Wi-A induced a marked increase in Rad51-PML colocalized foci in ALT cells at 4 h (Fig. 5D). By 24 h, these foci had nearly vanished, coincident with pronounced loss of Rad51. In contrast, no notable Rad51-PML colocalization was observed in TEP cells (Fig. 5D), even though Wi-A induced repairable DNA damage at 4 h (Fig. S4C) that recruited Rad51 to telomeres similarly to ALT cells (Fig. S5E, F). Importantly, PML knockdown completely rescued Rad51 depletion in Wi-A-treated ALT cells (Figs. 5E, F and S5G, H), restoring its accumulation at damaged telomeres (Figs. 5G and 2I) and attenuating the sensitivity of ALT cells to Wi-A (Fig. 5H). These findings highlight a PML-dependent mechanism responsible for Rad51 degradation specifically in ALT cells following Wi-A treatment.

### Wi-A destabilizes Rad51 in ALT cells by promoting its interaction with RFWD3

RFWD3, an E3 ubiquitin ligase, has been implicated in facilitating Rad51 ubiquitination at DNA damage sites, leading to its eventual degradation [44]. Interestingly, RFWD3 is also reported to be enriched in PML bodies in normal growing cells [45]. We therefore surmised that RFWD3 may play a role in the PML-dependent Rad51 degradation triggered by Wi-A-induced telomeric DNA damage in ALT cells.

**Fig. 6:**
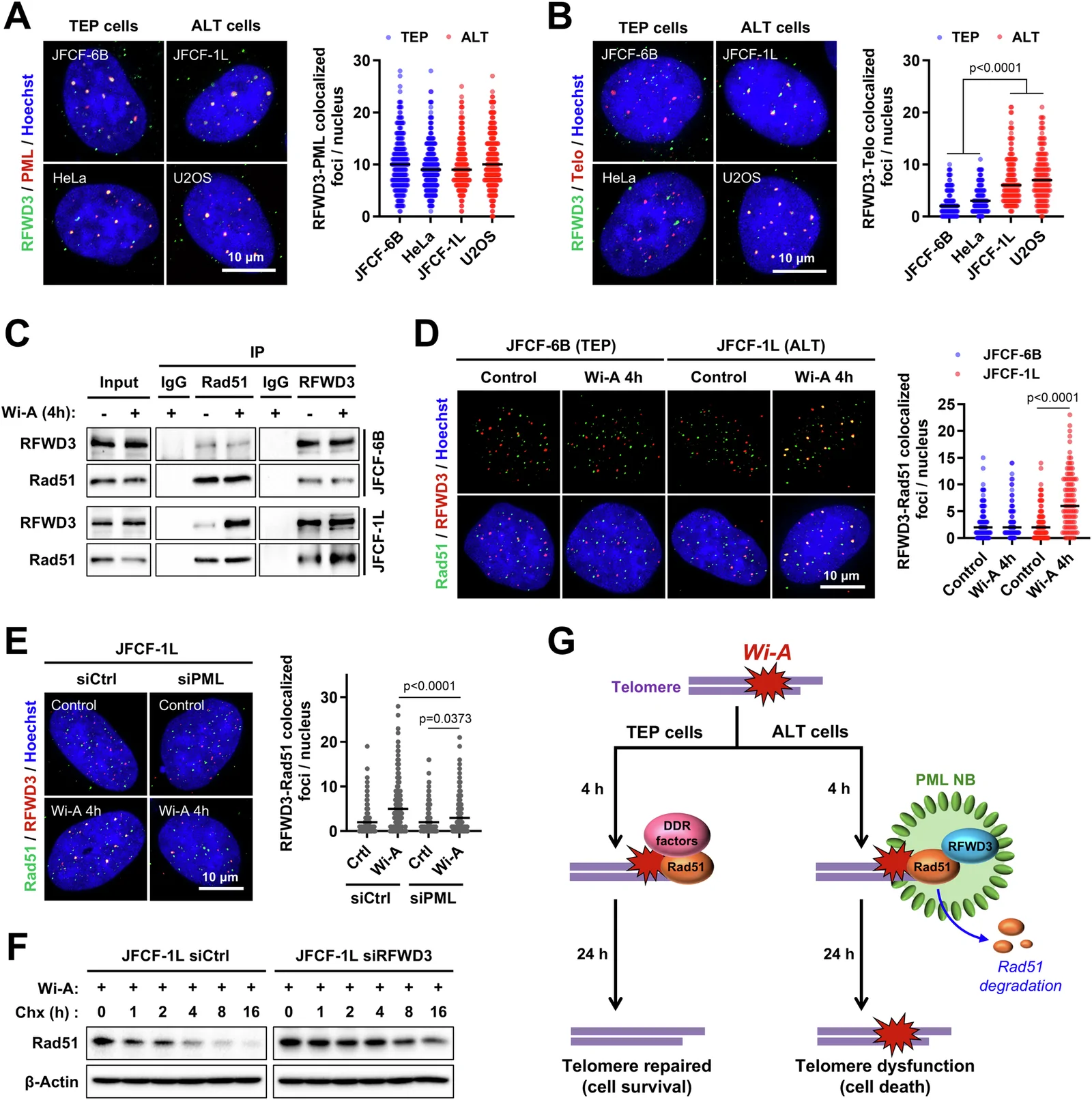
Wi-A destabilizes Rad51 in ALT cells by promoting its interaction with RFWD3. **A** Immunofluorescence of RFWD3 and PML showing their colocalization in both TEP and ALT cells. Quantification of RFWD3-PML foci per nucleus (*n* ≥ 200) is presented on the right. **B** Immunofluorescence showing RFWD3 colocalizes with telomeres exclusively in ALT cells. Quantification of RFWD3-telomere foci per nucleus (*n* ≥ 200) is shown on the right (one-way ANOVA test). **C** Co-immunoprecipitation of Rad51 and RFWD3 in TEP (JFCF-6B) and ALT (JFCF-1L) cells treated with or without Wi-A (0.5 μM for 4 h). An increased interaction between Rad51 and RFWD3 was observed in ALT cells after the treatment. **D** Representative images of Rad51 and RFWD3 staining showing increased colocalization in Wi-A-treated (0.5 μM for 4 h) ALT cells. Quantification of Rad51-RFWD3 foci per nucleus (*n* ≥ 100) is shown on the right (one-way ANOVA test). **E** PML compromised ALT cells showed attenuation of Wi-A-induced Rad51-RFWD3 colocalization. JFCF-1L cells transfected with control or PML siRNAs were treated with Wi-A (0.5 μM) for 4 h and subjected to immunostaining with Rad51 and RFWD3 antibodies. Quantification of Rad51-RFWD3 foci per nucleus (*n* ≥ 200) is shown on the right (one-way ANOVA test). **F** RFWD3 knockdown stabilized Rad51 in Wi-A treated ALT cells. JFCF-1L cells transfected with control or PML siRNAs were treated with Wi-A (0.5 μM) in combination with CHX (10 μg/mL) for indicated times and immunoblotted with Rad51 antibody. **G** Exposure to a moderate dose (0.5 μM) of Wi-A provokes DNA lesions at telomeres. In TEP cells, this damage rapidly (≤4 h) recruits DNA damage response (DDR) factors, including Rad51, enabling telomeric DNA repair within 24 h and ensuring cell survival. In ALT cells, telomeres specifically colocalize with PML nuclear bodies (PML NBs), where the E3 ubiquitin ligase RFWD3 is enriched. Following telomere damage, Rad51 is recruited to this complex and degraded through its interaction with RFWD3. This dysregulation of Rad51 leads to ALT telomere dysfunction, ultimately causing ALT cell death.

We performed immunostaining to analyze RFWD3 colocalization with PML and telomeres. Both TEP and ALT cells showed marked colocalization of RFWD3 and PML (Fig. 6A). However, a substantial number of RFWD3-telomere colocalized foci were observed exclusively in ALT cells (Fig. 6B), suggesting the presence of RFWD3 in ALT-associated PML bodies (APBs). We next tested whether RFWD3 interacts with Rad51 during the early phase of Wi-A treatment. Reciprocal immunoprecipitation showed an interaction between RFWD3 and Rad51, which was notably enhanced by 4 h treatment of Wi-A in ALT cells, but not in TEP cells (Fig. 6C). Immunostaining further supported these findings, revealing an increase in RFWD3-Rad51 colocalized foci specifically in Wi-A-treated ALT cells (Fig. 6D). Notably, Rad51 also colocalized with PML under the same conditions (Fig. 5D), suggesting the interaction between RFWD3 and Rad51 likely occurred in PML bodies. Furthermore, the observation that the increased RFWD3-Rad51 colocalized foci were partially diminished in ALT cells lacking PML confirmed the involvement of PML in mediating RFWD3-Rad51 binding in response to Wi-A (Fig. 6E). To assess the necessity of RFWD3 for Wi-A-induced Rad51 degradation, we knocked down RFWD3 using siRNA (Fig. S6A) and examined Rad51 stability in ALT cells following Wi-A treatment. The results showed that RFWD3 knockdown drastically prolonged the half-life of Rad51 (Figs. 6F and S6B), indicating its essential role in this degradation process. Collectively, these results provide evidence that the Rad51 degradation in Wi-A-treated ALT cells results from the enhanced interaction between Rad51 and RFWD3 within PML bodies.

## Discussion

While telomerase inhibitors have garnered more attention due to the prevalence of telomerase activation in 80-90% of cancers, targeting the ALT mechanism remains a substantial clinical challenge. Compounding the challenge, the coexistence of telomerase and ALT has been observed within individual tumors, with the inhibition of one mechanism potentially activating the other [46, 47]. This interdependence underscores the necessity for therapeutic drugs against ALT cancers specifically. The natural compound Wi-A emerges as a promising candidate, as it preferentially induces apoptosis in ALT cells [28]. Here, using a cDNA array-based transcriptomic screen in an isogenic pair of TEP and ALT cells, we observed that Wi-A exerted a stronger inhibitory effect in ALT cells on genes involved in PI3K/AKT signaling (Fig. 1A, B), a pathway previously implicated in maintaining genomic stability through regulating telomere integrity [48, 49]. Notably, analysis of untreated isogenic TEP and ALT cells revealed no substantial differences in the expression of PI3K/AKT pathway proteins (Fig. S7A), indicating that the differential response to Wi-A is unlikely to arise from baseline disparities between the two cell types. PTEN acts as an upstream inhibitor of the PI3K/AKT pathway, and its deficiency, along with constitutive activation of PI3K/AKT signaling, is observed in approximately 40% of tumors that are frequently ALT-positive, such as glioblastoma [50, 51] and osteosarcoma [52]. Given that ALT telomeres are characterized by genomic instability, including elevated replication stress and DNA damage signaling [53], ALT cells may therefore rely more heavily on PI3K/AKT signaling to cope with endogenous genomic stress. The increased PTEN protein levels (Fig. 1C) and reduced proliferation (Fig. S2A, B) observed to a greater extent in Wi-A-treated ALT cells further support an ALT-specific effect of Wi-A. In addition, Wi-A pre-treatment followed by UV irradiation induced dense and fragmented 53BP1 foci in ALT cells, a phenotype not observed in TEP cells (Fig. 3B). This morphological alteration is consistent with patterns reported in camptothecin-treated ALT (U2OS) versus TEP (MDA-MB-231) cells that exhibit divergent cell-fate outcomes [54]. In line with this, we observed defective coordination of DNA repair specifically in JFCF-1L ALT cells upon Wi-A treatment (Figs. 3E and S3). Collectively, our results suggest Wi-A may sensitize ALT cells to PI3K/AKT-associated replication stress and impaired DNA repair capacity, resulting in ALT-specific telomere dysfunction (Figs. 2 and 3).

DNA replication stress is closely associated with stalled replication forks, which trigger spontaneous telomeric DNA synthesis in ALT cells [55]. However, without timely repair by DDR factors, these stalled forks can collapse, resulting in DSBs [56]. At ALT telomeres, Rad51 recruitment is essential for resolving replication-associated DNA damage, thereby supporting cell survival by preventing fork collapse [57]. Our molecular docking analysis showed that Wi-A does not disrupt the Rad51-BRCA2 complex (Fig. S4A). Instead, it occupies the ATP-binding pocket at the Rad51 protomer-protomer interface, leading to Rad51 filament destabilization (Fig. 4A–C, and Table S1). This was corroborated by the marked loss of Rad51 foci in cisplatin-stressed cells exposed to Wi-A (Fig. 4G, H). Notably, a rapid reduction in Rad51 levels was observed in ALT cells as early as 4 h following Wi-A treatment, at which time no detectable effects on cell proliferation were evident (Fig. S7B), indicating an early and direct effect of Wi-A on Rad51 stability. Consistent with this, both siRNA-mediated and pharmacological inhibition of Rad51 recapitulated key phenotypes observed in Wi-A-treated ALT cells, including DSBs-associated growth arrest (Fig. S7C) and increased sensitivity to DNA-damaging agent and IR (Fig. S7D), supporting a role for Rad51 as a functional target of Wi-A. Interestingly, although Rad51-mediated HR was also modestly reduced in TEP cells following Wi-A treatment (Fig. 3I), these cells still resolved DNA damage efficiently (Fig. 3E), implying the involvement of HR-independent repair pathways.

A hallmark of ALT cells is the clustering of telomeres within PML bodies, forming APBs that scaffold DNA repair factors such as Rad51 and RPA [58]. We observed that Wi-A treatment induced transient Rad51-PML colocalization at telomeric damage foci, followed by pronounced loss of Rad51 foci over 24 h (Fig. 5D). PML depletion rescued Rad51 from degradation and mitigated Wi-A cytotoxicity (Fig. 5E–H), revealing a PML-dependent mechanism. Notably, the E3 ligase RFWD3, which mediates Rad51 ubiquitination [44], also localizes to PML bodies, particularly in S/G2 when HR is active [45, 59, 60]. Wi-A-induced G2 arrest in ALT cells (Fig. S2B) may further favor this spatial proximity. Given that PML bodies serve as hubs for proteasomal degradation and engage multiple ubiquitin ligases [20, 42, 61], we propose that APBs likely act as regulatory sites where unrepaired telomeres, Rad51, and RFWD3 converge. Within this context, Wi-A enhances RFWD3-Rad51 interaction and promotes Rad51 degradation (Fig. 6C, D), thereby dismantling HR repair machinery at ALT telomeres and sensitizing ALT cells to the treatment.

While our results delineate a clear mechanistic model of distinct responses of TEP and ALT cells to Wi-A treatment in vitro (Fig. 6G), in vivo validation is warranted. Moreover, the exact mechanism by which RFWD3 is recruited to APBs is unclear. Given that RFWD3 is known to interact with RPA2 at DNA damage-induced foci [44, 62, 63], we speculate that RPA2 may serve as a mediator facilitating the formation of an RFWD3-RPA2-Rad51 complex at Wi-A-damaged telomeres within PML bodies, positioning Rad51 for RFWD3-dependent degradation. Future studies should investigate this possibility and assess Wi-A efficacy in animal models to confirm its ALT-specific therapeutic potential.

In summary, our findings establish Wi-A as a promising therapeutic agent that selectively targets the ALT pathway by disrupting Rad51-dependent HR repair. Through a PML- and RFWD3-mediated mechanism, Wi-A induces degradation of Rad51, leading to exacerbated replication stress and persistent telomeric damage exclusively in ALT cells. This selective vulnerability highlights the therapeutic potential of Wi-A as a natural compound for targeting cancers that rely on the ALT mechanism for telomere maintenance.

## Materials and methods

### Cell lines, culture and treatments

Human osteosarcoma (U2OS and Saos-2), breast carcinoma (MCF7) and cervical adenocarcinoma (HeLa) were purchased from Japanese Collection of Research Bioresources Cell Bank (JCRB Cell Bank; Osaka, Japan). SV40-immortalized TEP (JFCF-6/T.1J/6B (JFCF-6B), JFCF-6/T.1J/6G (JFCF-6G)) and ALT (JFCF-6/T.1L, (JFCF-1L), JFCF-6/T.1J/1-4D (JFCF-4D)) cells were derived from jejunal parental fibroblasts (JFCF-6), as previously described [64–66]. The cells were authenticated by the source and cultured in Dulbecco’s modified Eagle’s medium (DMEM; Gibco BRL, Grand Island, NY, USA) supplemented with 10% fetal bovine serum (Gibco BRL) at 37 °C in a humidified incubator containing 5% CO_2_.

Treatments with Wi-A (Wako, Osaka, Japan), RI-1 (Selleckchem, Houston, TX, USA), cisplatin (Sigma-Aldrich, St. Louis, MO, USA), or MG132 (Sigma-Aldrich) were conducted when cell confluency reached about 50%. UV and X-ray irradiation were performed using a UV crosslinker (FB-UVXL-1000; Fisher Scientific, Loughborough, UK) and an X-ray generator (CP-160; Faxitron, AZ, USA), respectively. Cells were irradiated in PBS and either fixed or harvested immediately, or allowed to recover under standard culture conditions prior to analysis. Negative control siRNA (catalog #4390843), RML siRNA (catalog #4390824), RFWD3 siRNA (catalog #4392420) and Rad51 siRNA (catalog #4392420) were obtained from Thermo Fisher Scientific (Waltham, MA, USA). siRNA transfections were carried out with Lipofectamine RNAiMAX (Thermo Fisher Scientific), following the manufacturer’s protocols.

### Cell proliferation assay

Cells were seeded in 6-well plates (3 × 10⁵ cells per well, in triplicate) and counted at indicated time points after Wi-A treatment using a TC20 Automated Cell Counter (Bio-Rad, Hercules, CA, USA). Viable cells were determined by trypan blue exclusion. Growth curves were generated from three independent experiments.

For colony formation assays, control or siRNA-transfected cells were seeded in 12-well plates (500 cells per well, in triplicate), allowed to adhere overnight, and treated with or without Wi-A as indicated. Cells were maintained under standard conditions with medium replaced every 3 days until colonies were formed. Colonies were washed with cold PBS, fixed with methanol/acetone (1:1, v/v), and stained with 0.1% crystal violet (Wako).

### EdU labeling

EdU (5-ethynyl-2′-deoxyuridine) was visualized using a Click-iT Plus EdU imaging kit (Invitrogen, Carlsbad, CA, USA) according to the manufacturer’s recommendations. Briefly, cells were plated in 12-well plates at a density of 8 × 10^4^ cells/well. After treatment with Wi-A for 24 h, cells were washed with PBS thrice and incubated in EdU-containing medium for 1 h. The cells were then fixed with 4% paraformaldehyde, permeablised with 0.2% Triton X-100 in PBS, and labeled with Click-iT Alexa Fluor 488 dye and Hoechst 33342, as provided in the kit.

### **Flow cytometry analysis**

Control and treated cells were detached by trypsin, washed with cold PBS, and added dropwise to pre-chilled 70% ethanol. The fixed cells were centrifuged at 500 × *g* for 5 min at 4 °C, washed with cold PBS twice, followed by resuspension in 1 mL cold PBS. 100 μg/ml RNaseA (Thermo Fisher Scientific) was added to the cell suspensions at 37 °C for 1 h followed by centrifugation (500 × *g*) at 4 °C for 5 min. Cells were resuspended in 200 μL of Guava Cell Cycle reagent (Millipore, Billerica, MA, USA), incubated for 30 min at room temperature away from light and analyzed by Guava PCA flow cytometer (Millipore). The data were subsequently analyzed using the FlowJo software (Ashland, OR, USA).

### **Western blotting**

Whole-cell lysate was prepared using RIPA buffer (Thermo Fisher Scientific) supplemented with protease and phosphatase inhibitor cocktails (Roche, Mannheim, Germany). Nuclear and cytoplasmic fractions were prepared using the NE-PER® Nuclear and Cytoplasmic Extraction reagents (Thermo Fisher Scientific) as per the manufacturer’s instructions. The lysates (20 μg) were separated by SDS-PAGE and transferred to a polyvinylidene difluoride (PVDF) membrane (Millipore) by electro-blotting. Following 1 h blocking with 3% bovine serum albumin (Wako) at room temperature, membranes were probed with target protein-specific primary antibodies (detailed in Supplementary Table S2) overnight at 4 °C. The immunoblotted membranes were incubated with horse-radish peroxidase (HRP)-conjugated secondary antibodies for 2 h at room temperature and detected by enhanced chemiluminescence reaction. Quantitative analysis of immunoreactive bands was performed using ImageJ (National Institute of Health, Bethesda, MD, USA).

### Co-immunoprecipitation

Dynabeads Protein A Immunoprecipitation Kit (Invitrogen) was used according to the manufacturer’s protocol. Briefly, cells were lysed on ice for 30 min in Pierce™ IP Lysis Buffer (Thermo Fisher Scientific) supplemented with a protease inhibitor cocktail (Roche). The lysates were centrifuged at 13,000 rpm for 15 min at 4 °C, and the supernatant was collected. Protein concentration was determined using the Pierce™ BCA Protein Assay Kit (Thermo Fisher Scientific). Magnetic beads were pre-incubated with the appropriate primary antibody at room temperature for 1 h and subsequently mixed with the protein lysates for overnight rotation at 4 °C. The next day, the beads were washed several times with PBS containing 0.05% Tween 20, and the bound proteins were eluted by incubating the beads in sample buffer at 70 °C for 15 min. Eluates were then analyzed by immunoblotting.

### Immunostaining

Cells were seeded on glass coverslips in a 12-well culture dish and allowed to adhere overnight. Following the indicated treatments, cells were treated with a pre-extraction buffer (0.1% Triton X-100, 20 mM HEPES-KOH pH 7.9, 50 mM NaCl, 300 mM sucrose) for 5 min on ice to remove unbound chromatin proteins and fixed with 4% paraformaldehyde for 10 min. The fixed cells were then washed with PBS, permeabilized with 0.5% Triton X-100 in PBS for 10 min, blocked with 2% bovine serum albumin (BSA) in PBS for 1 h, and incubated with primary antibodies at 4 °C overnight. After extensive washing with 0.2% Triton X-100 in PBS, cells were incubated with Alexa-594/488-conjugated secondary antibodies and counterstained with Hoechst 33342 (Sigma-Aldrich) for 10 min in dark. A detailed list of antibodies used in this study is shown in Supplementary Table S2. The coverslips were mounted on glass slides and visualized using a Carl Zeiss Axio Observer microscope (Tokyo, Japan). Images were captured across multiple Z-sections, deconvoluted, and converted into 2D maximum projection images using ZEN software (Carl Zeiss). Foci counting and colocalization analysis were performed with the ComDet plugin in ImageJ.

### Comet assay

DNA double-strand breaks were detected using Trevigen’s comet assay system (Gaithersburg, MD, USA) under neutral conditions, following the manufacturer’s instructions. Briefly, cells were embedded in low-melting-point agarose and spread onto glass slides. After solidification, the slides were incubated overnight in lysis buffer, followed by neutral electrophoresis and refrigerated electrophoresis. After sequential washes, slides were stained with GelGreen (Biotium, Hayward, CA, USA), and migrating DNA fragments were visualized using a fluorescence microscope (Axio Observer, Carl Zeiss, Tokyo, Japan). For each condition, at least 200 randomly selected cells were analyzed. The percentage of DNA in the tail was quantified using OpenComet v1.3, a plugin for ImageJ.

### **Homologous recombination assay**

JFCF TEP and ALT cells stably expressing HR reporter DR-GFP (Addgene #26475) were established by G418 (Sigma-Aldrich) selection. These cells were subsequently transfected with the pCBASceI plasmid (Addgene #26477) using FuGENE HD (Promega, Madison, WI, USA). Briefly, 3 × 10^5^ DR-GFP-expressing cells were transfected with 2 μg I-SceI–expressing plasmid in a 6-well plate. After 24 h, cells were washed and allowed to grow for an additional 24 h in the fresh growth medium in the presence or absence of 0.25 μM Wi-A. The Rad51 inhibitor RI-1 was used for comparison at a concentration of 5 μM. Cell lysates were harvested 48 h after transfection and subjected to a microplate reader (Infinite M200 Pro; Tecan, Männedorf, Switzerland) to determine the restored GFP intensity. Relative HR efficiency was calculated as the mean of GFP fluorescence.

### Luciferase reporter assay

Cells were co-transfected with reporter constructs (PG13-luc, Addgene #16442, or WWP-Luc, Addgene #16451) and the pRL-TK control reporter vector (Promega) using FuGENE HD (Promega). After 24 h of Wi-A treatment, luciferase activity was measured using the Dual-Luciferase Reporter Assay System (Promega), according to the manufacturer’s instructions. Results are expressed as the ratio of firefly to Renilla luminescence intensity. Experiments were performed in triplicate and repeated at least three times.

### RNA isolation and qRT-PCR

Total RNA was isolated from cells using the RNeasy mini kit (Qiagen, Standford Valencia, CA, USA) following the manufacturer’s protocol. All RNA samples were treated with RNase-free DNase I (Promega) and quantified using a Nanodrop spectrophotometer (NanoDrop One; Thermo Fisher Scientific). cDNA was synthesized following the protocol of QuantiTect Reverse Transcription Kit (Qiagen). Real-time RT-PCR was performed using SYBR® Select Master Mix (Thermo Fisher Scientific) on Eco™ real time system (Illumina, San Diego, CA, USA). Relative mRNA levels were quantified using the comparative △Ct method by normalizing to the endogenous reference gene and expressed as fold changes. Supplementary Table S3 shows the primer sequences used in this study.

### Cycloheximide chase assay

Cells were cultured in medium containing 10 μg/mL cycloheximide (Sigma-Aldrich) to inhibit protein synthesis, with or without 0.5 μM Wi-A. At the indicated time points post-cycloheximide treatment, cells were harvested, and whole-cell lysates were prepared for immunoblot analysis. For siRNA-transfected cells, the treatment was initiated 24 h after transfection.

### ATP assay

Cellular ATP levels were measured using a luminescent ATP detection assay kit (Abcam, Cambridge, UK) according to the manufacturer’s protocol. Briefly, 4000 cells per well were plated in a 96-well plate and exposed to Wi-A at 0.5 μM for 24 h. Substrate buffer was then added for luminescence detection on a microplate reader (Infinite M200 Pro; Tecan). The relative ATP levels were expressed as fold change of JFCF-6B control.

### Microarray and transcriptome analysis

The total RNA was reverse transcribed and labeled using a low input Quick Amp Labeling Kit (Agilent Technologies, Santa Clara, CA) according to the manufacturer’s instructions, and then hybridized to a GeneChip Mouse Gene 1.0 ST array (Affymetrix). The raw fluorescence intensity of each probe was quantified using GeneChip Command console software (Affymetrix). The signal values obtained from the control and Wi-A-treated cells were normalized to the individual median of fluorescent intensity using GeneSpringGX software (Agilent Technologies). The change in expression of different genes in normalized samples compared with normalized control was calculated and expressed as log2-fold change. The Mfuzz R package (http://mfuzz.sysbiolab.eu/) was used to analyze the expression dynamics of genes in cells upon Wi-A treatment, and all genes were categorized into nine clusters based on their expression patterns. The genes in cluster 4 and 9 were subjected to Kyoto Encyclopedia of Genes and Genomes (KEGG) enrichment analysis using KOBAS (http://bioinfo.org/kobas) based on Over Representation Analysis (ORA), and the top 30 significantly (corrected *P*-value < 0.05) enriched pathways were displayed. The gene set enrichment analysis (GSEA) was operated using the clusterProfiler R package (https://bioconductor.org/packages/release/bioc/html/clusterProfiler.html).

### **Molecular docking**

Crystal structures of the Rad51-BRCA2 complex (PDB Id: 1N0W) and Rad51-ATP filament (PDB Id: 5NWL) were obtained from the Protein Data Bank. The structure of the Rad51-BRCA2 complex and the Rad51-Rad51 protomer complex were prepared using the PrepWizard module of Schrodinger suite 2018-4. The structure of the ligand, Wi-A, was retrieved from the PubChem database and prepared using the LigPrep module of Schrodinger suite [67]. Glide Extra Precision (XP) algorithm was used to perform docking of Wi-A at the interaction interface of the Rad51-BRCA2 complex and the ATP binding site of the Rad51-Rad51 protomer interface [68]. The docked complexes were simulated to monitor the stability of the ligand-bound complexes using Desmond module of Schrodinger suite. The protein-ligand complexes were simulated in an Optimized Potential for Liquid Simulations 3 (OPLS3) force field in a TIP4P solvated periodic box with 10 Å spacing. The solvation of the complexes was followed by neutralization, minimization for up to 2000 iterations. Minimized system is heated upto 300 K, equilibrated and simulated for a time period of 100 ns. Root mean square deviation (RMSD), hydrogen bonds analysis and conformational changes over the simulation trajectories of protein-ligand complexes were monitored using VMD version 1.9.4. The LigPlus program was used to study protein-ligand interactions. The protein-protein binding energy in the presence and absence of Wi-A was calculated using the PRODIGY web server [69]. Images for this publication were generated using PyMOL molecular graphics system (The PyMOL Molecular Graphics System, Version 2.0 Schrodinger, LLC, New York, NY, USA).

### Statistical analysis

The observed data are presented as the means ± SD, of at least three independent experiments. Pairwise differences were identified using Student’s *t* test and comparisons of multiple groups were performed using one-way ANOVA followed by Tukey test. A *P* value of 0.05 or less was considered significant. Statistical analyzes were performed using GraphPad Prism software (version 10.6.1; San Diego, CA, USA).

## Supplementary information

Supplementary Information

Uncropped Blots

## Data Availability

All data generated or analyzed during this study are included in this published article and its supplementary information files. Additional datasets supporting the conclusions of this study are available from the authors upon reasonable request. The supplementary information and full-length, uncropped western blot images are available at Cell Death & Disease’s website.
