# Supplementary material for: Selective killing of ALT-cancer cells by Withaferin-A involves destabilization of Rad51 and disruption of DNA repair
Source: Cell Death Dis. 2026 Jul 15;17(1):873. doi: 10.1038/s41419-026-09115-z (PMC13649652; doi:10.1038/s41419-026-09115-z)
Supplement: Supplementary file 1 [file 41419_2026_9115_MOESM2_ESM.pdf]

Figure 1C

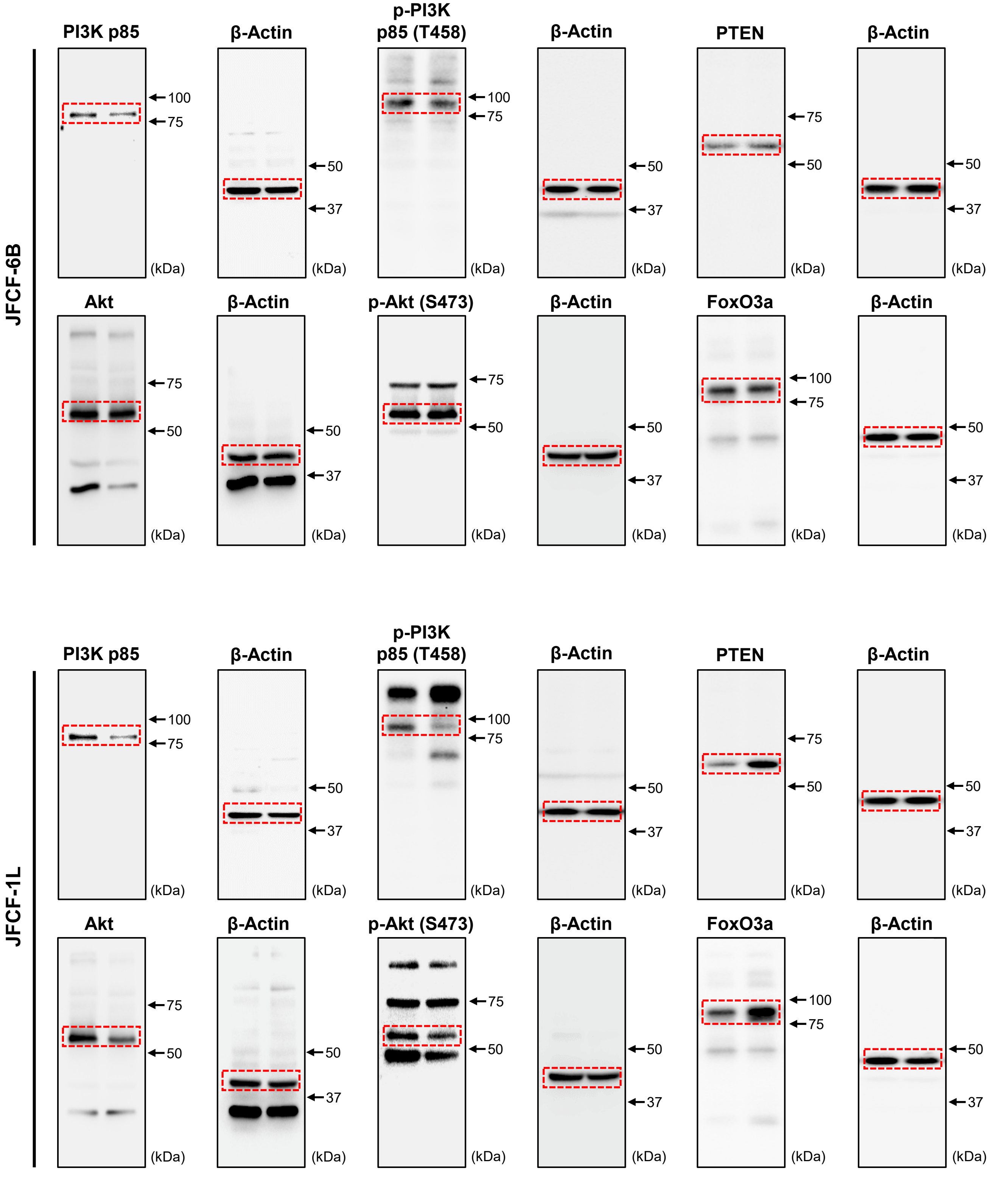
JFCF-6B
PI3K p85
β-Actin
p-PI3K p85 (T458)
β-Actin
PTEN
β-Actin
Akt
β-Actin
p-Akt (S473)
β-Actin
FoxO3a
β-Actin
JFCF-1L
PI3K p85
β-Actin
p-PI3K p85 (T458)
β-Actin
PTEN
β-Actin
Akt
β-Actin
p-Akt (S473)
β-Actin
FoxO3a
β-Actin
100
75
50
37
(kDa)

Figure 1D

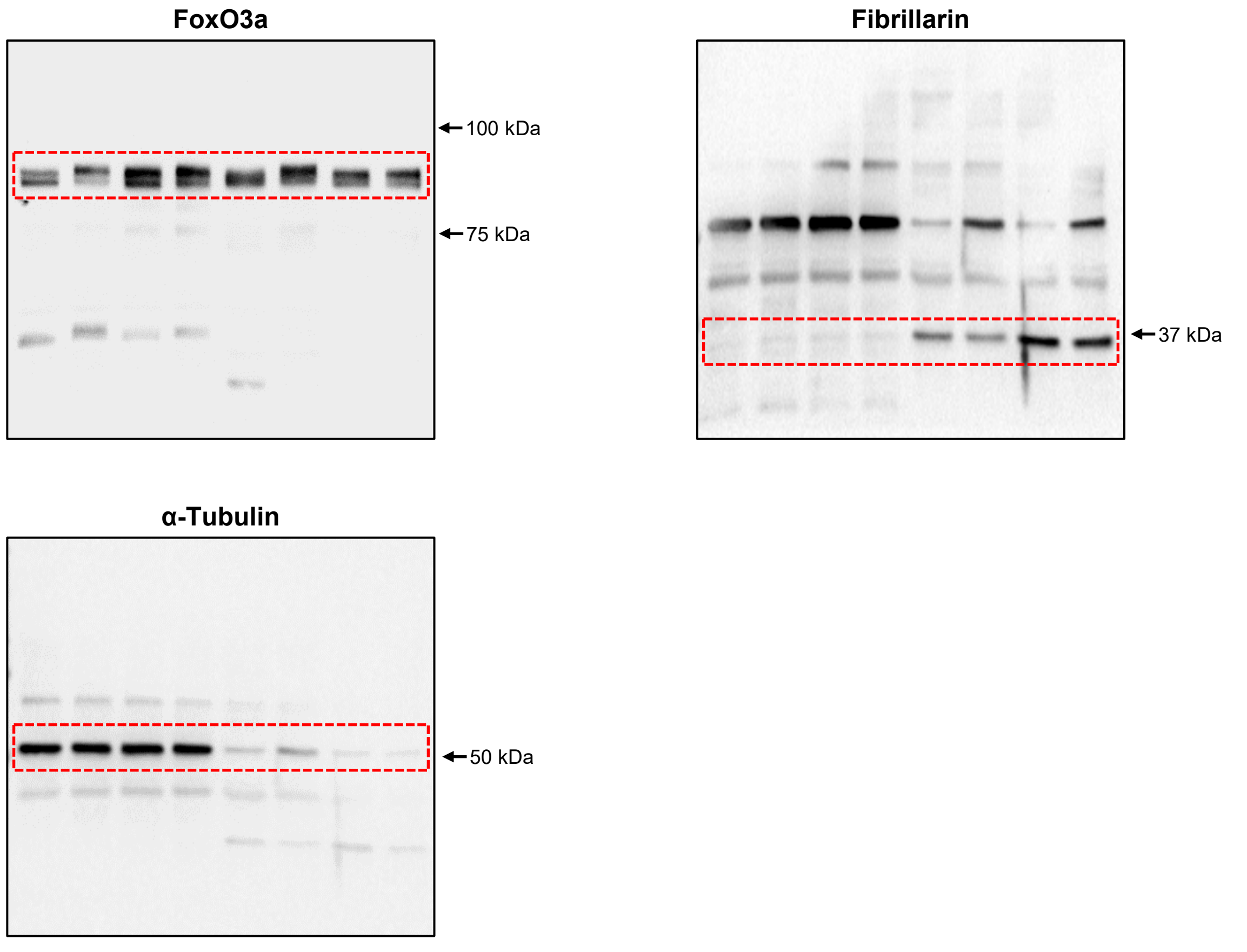

Figure 2B

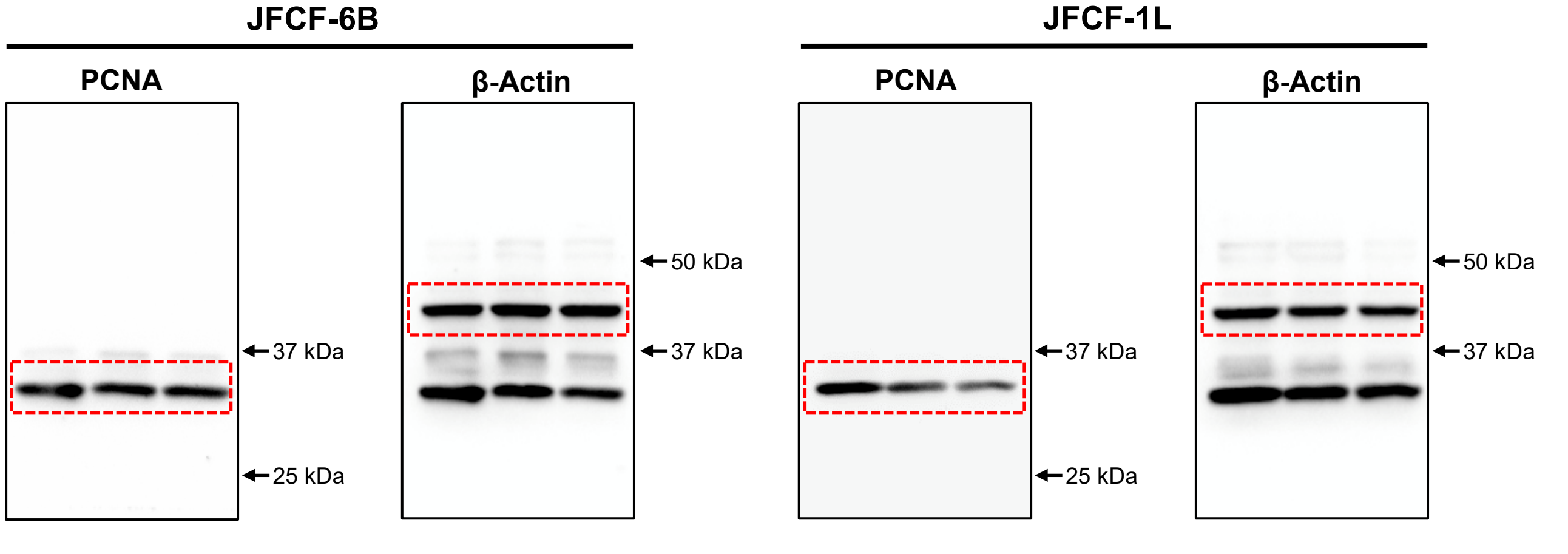

Figure 2D

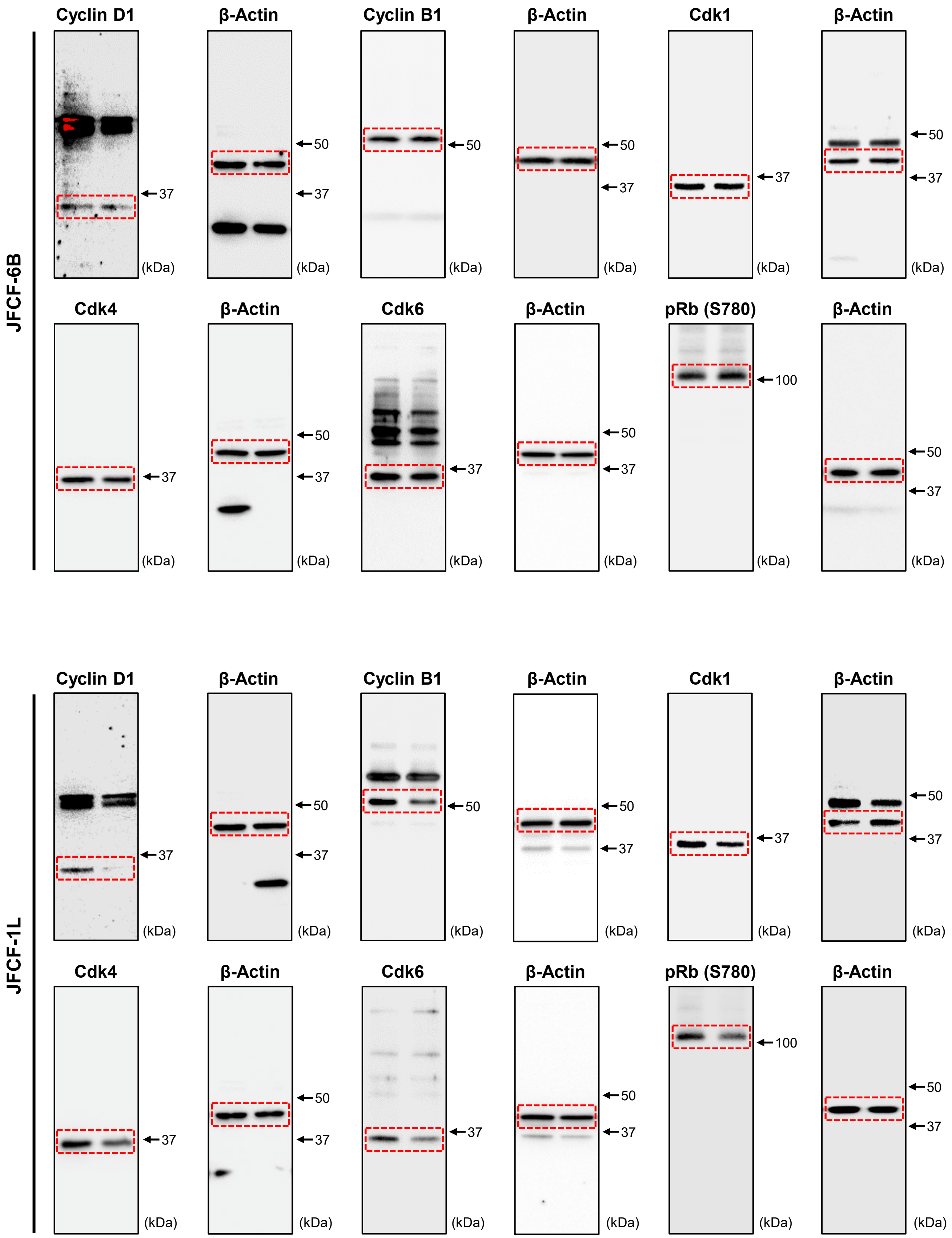
JFCF-6B
Cyclin D1
β-Actin
Cyclin B1
β-Actin
Cdk1
β-Actin
Cdk4
β-Actin
Cdk6
β-Actin
pRb (S780)
β-Actin
JFCF-1L
Cyclin D1
β-Actin
Cyclin B1
β-Actin
Cdk1
β-Actin
Cdk4
β-Actin
Cdk6
β-Actin
pRb (S780)
β-Actin
100
50
37
(kDa)

Figure 2F

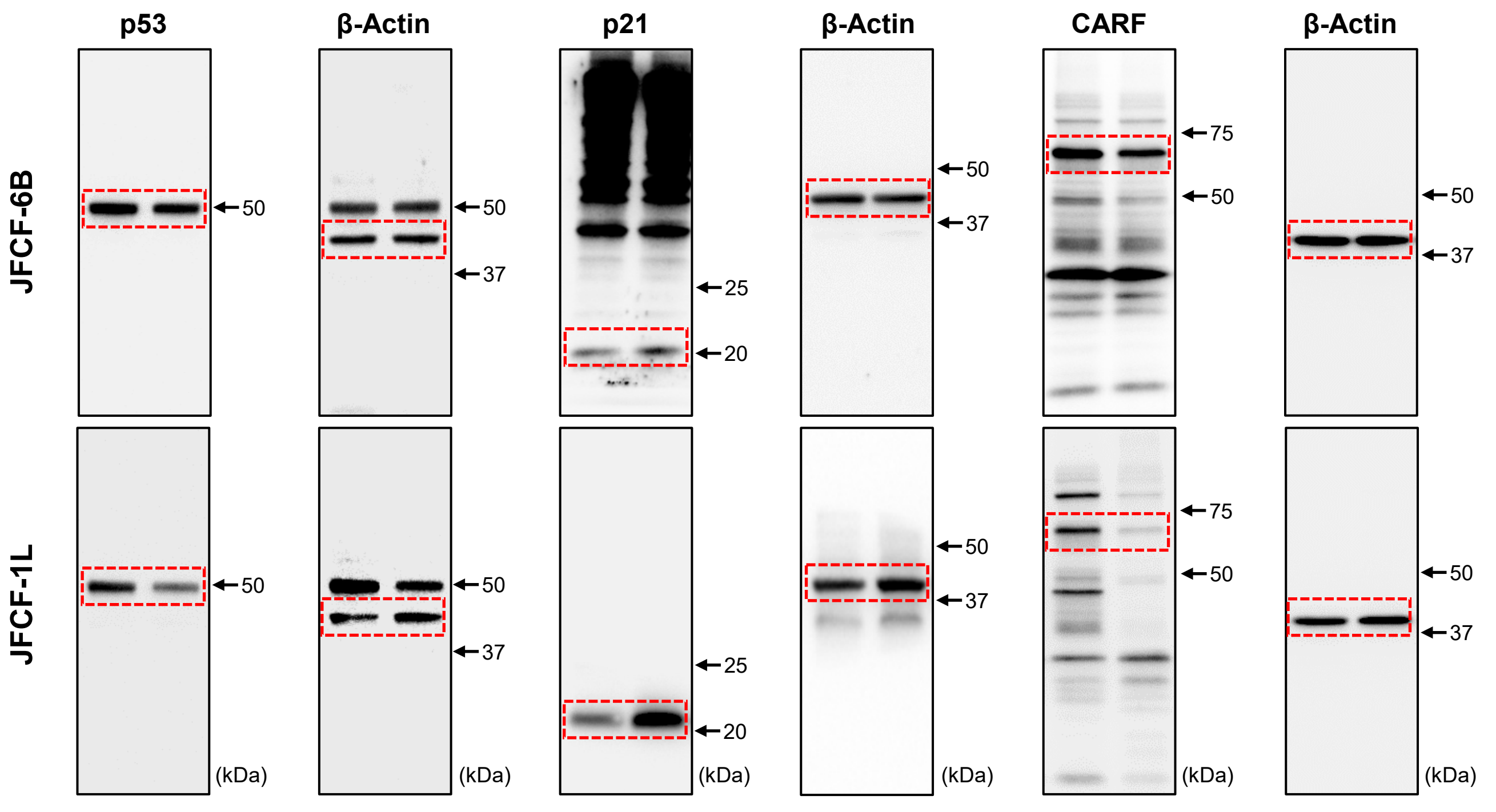

Figure 2G

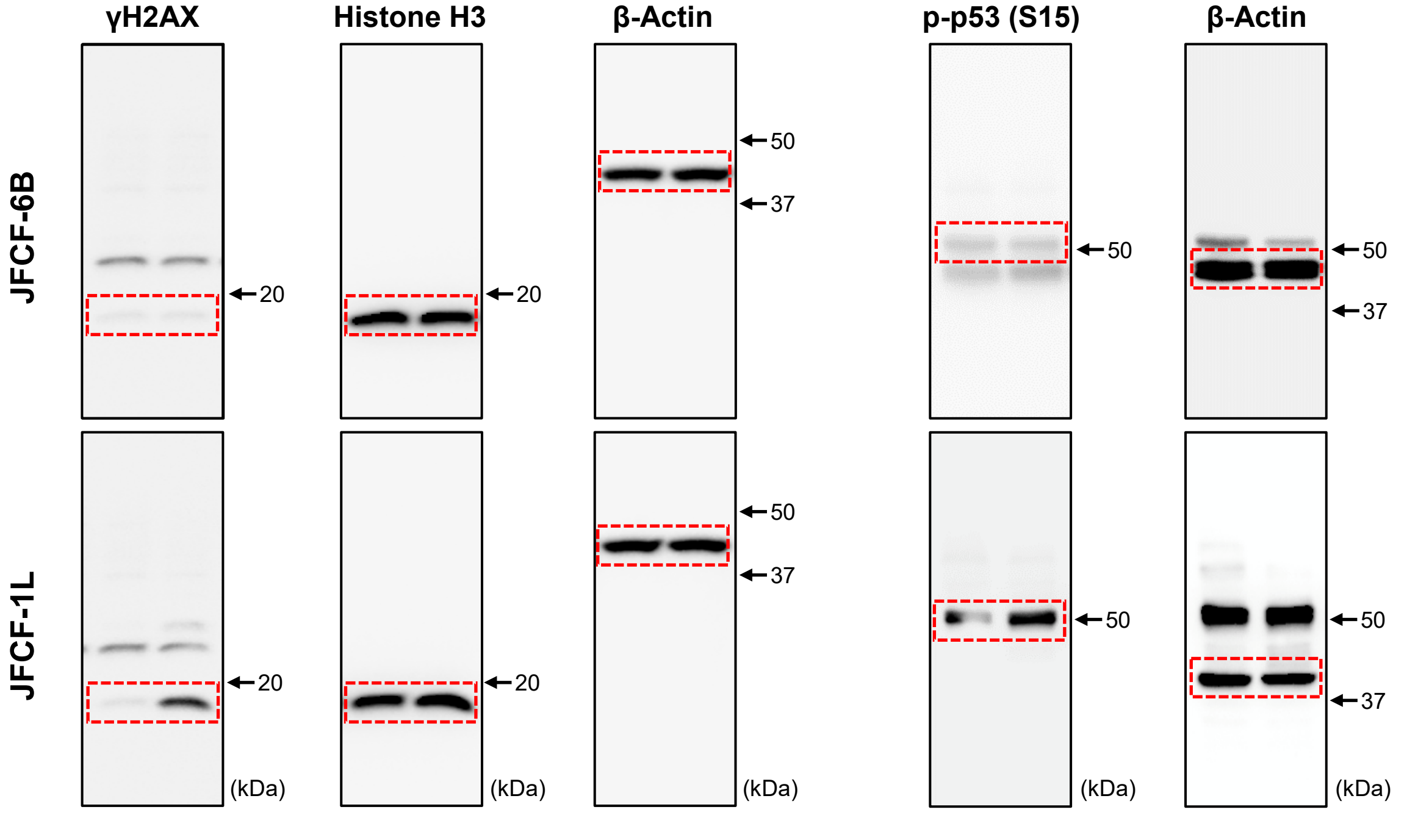

Figure 3E

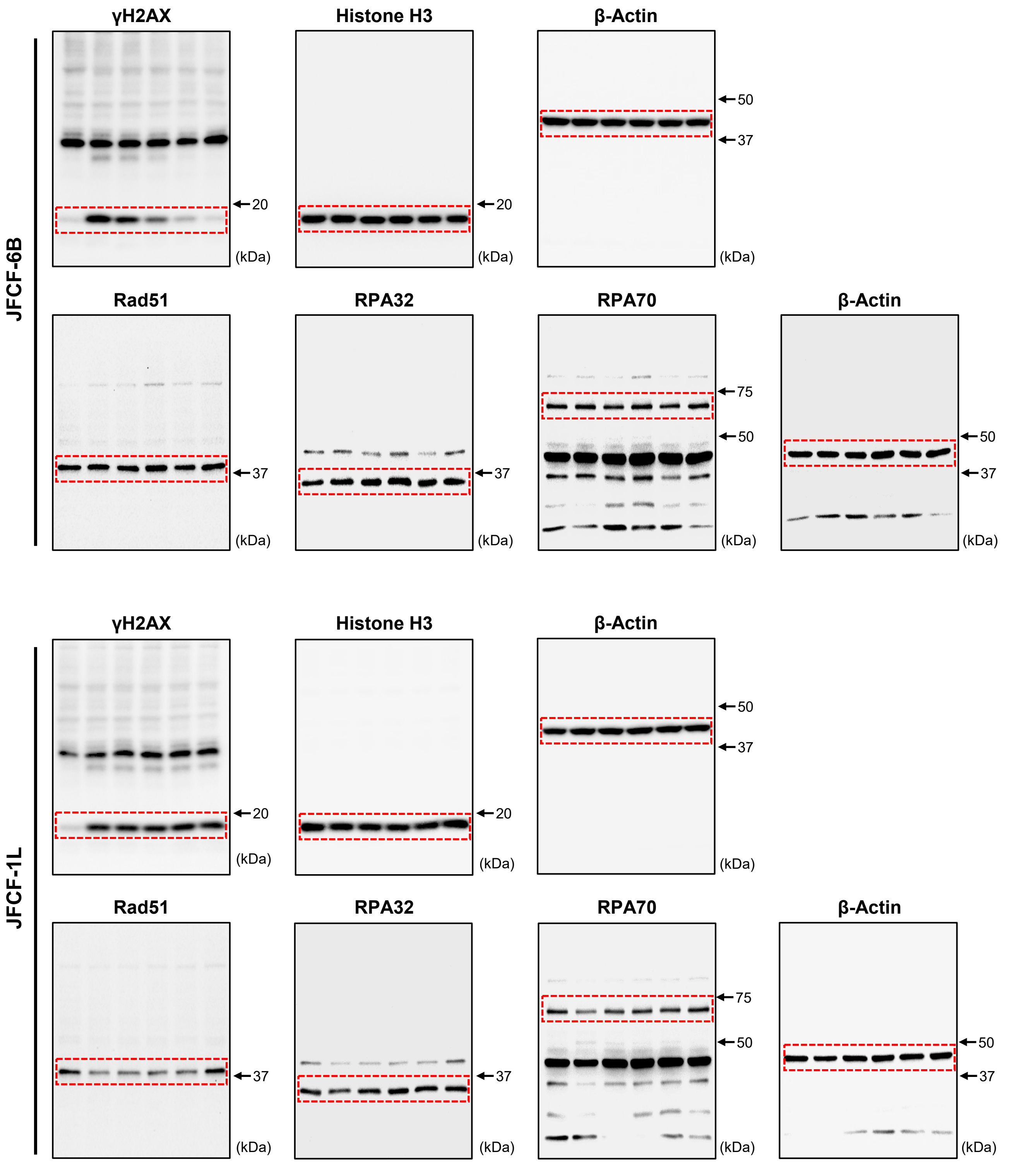
JFCF-6B
γH2AX
Histone H3
β-Actin
Rad51
RPA32
RPA70
β-Actin
JFCF-1L
γH2AX
Histone H3
β-Actin
Rad51
RPA32
RPA70
β-Actin
75
50
37
20
(kDa)

Figure S3A

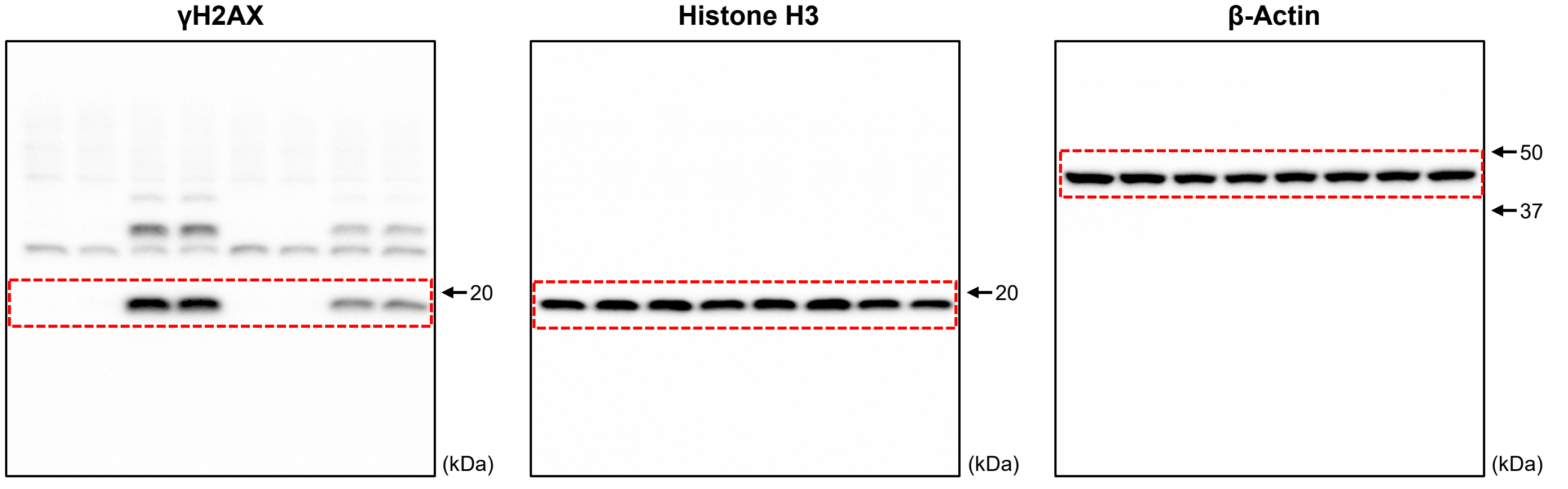

Figure S3B

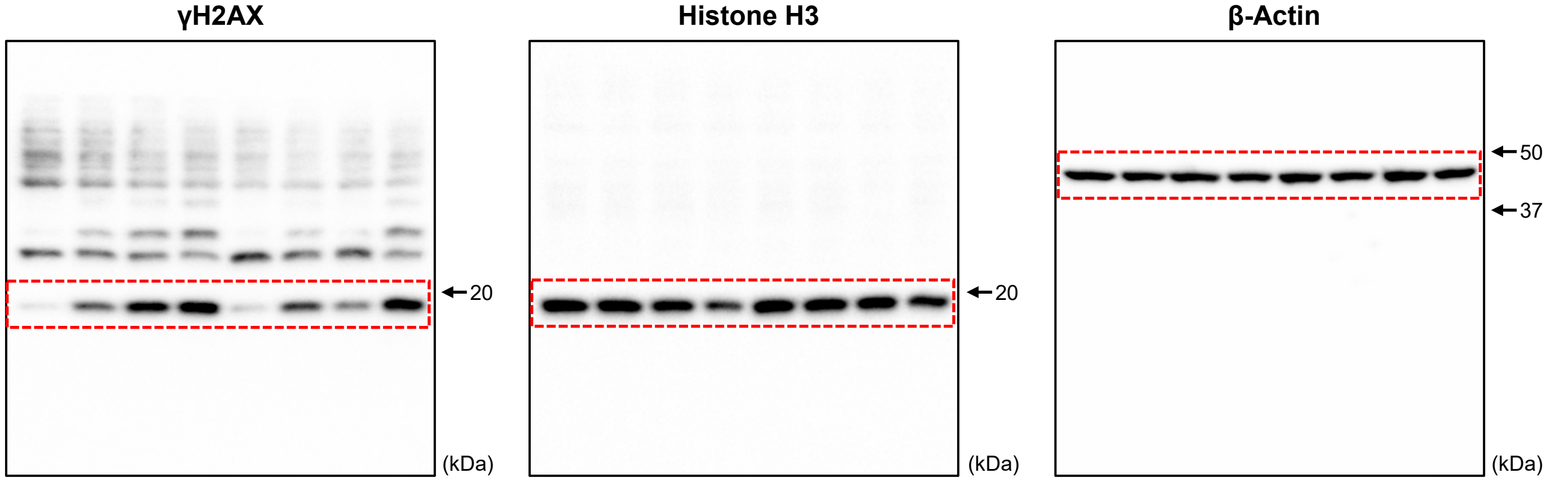

Figure S4C

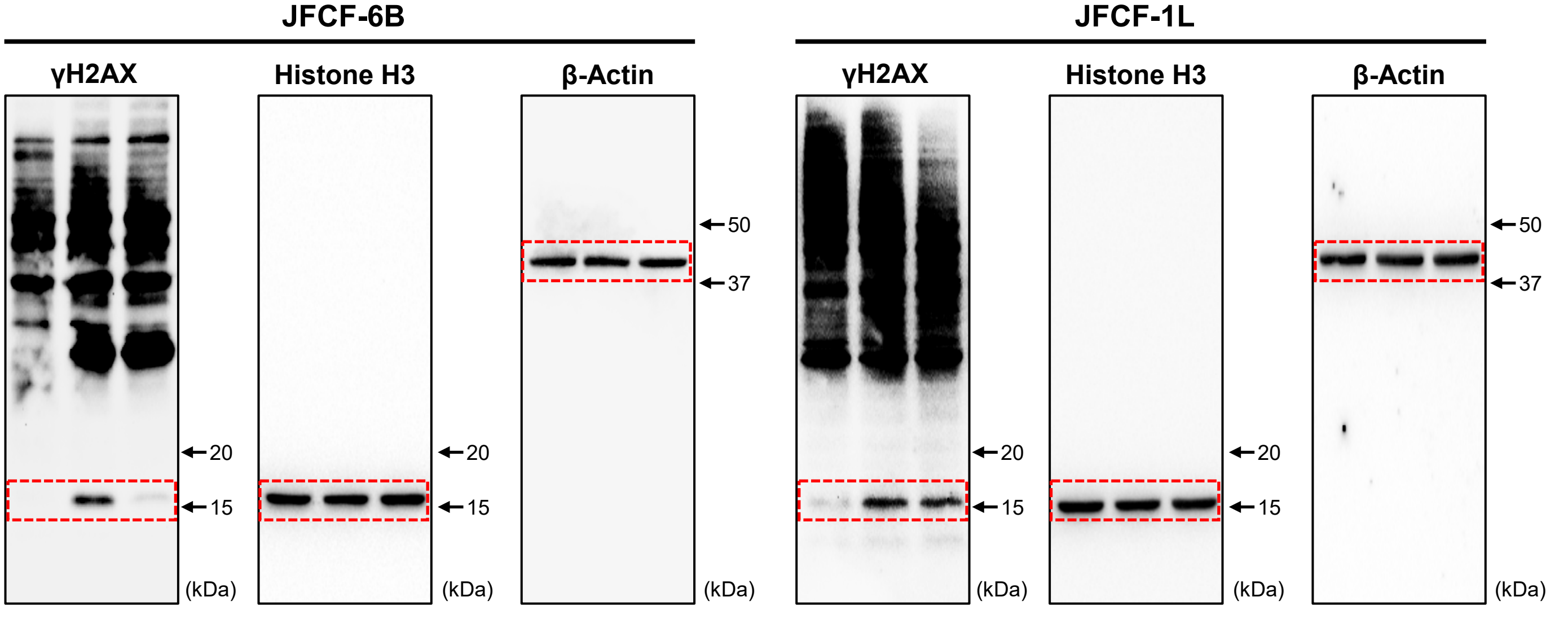

Figure 5A

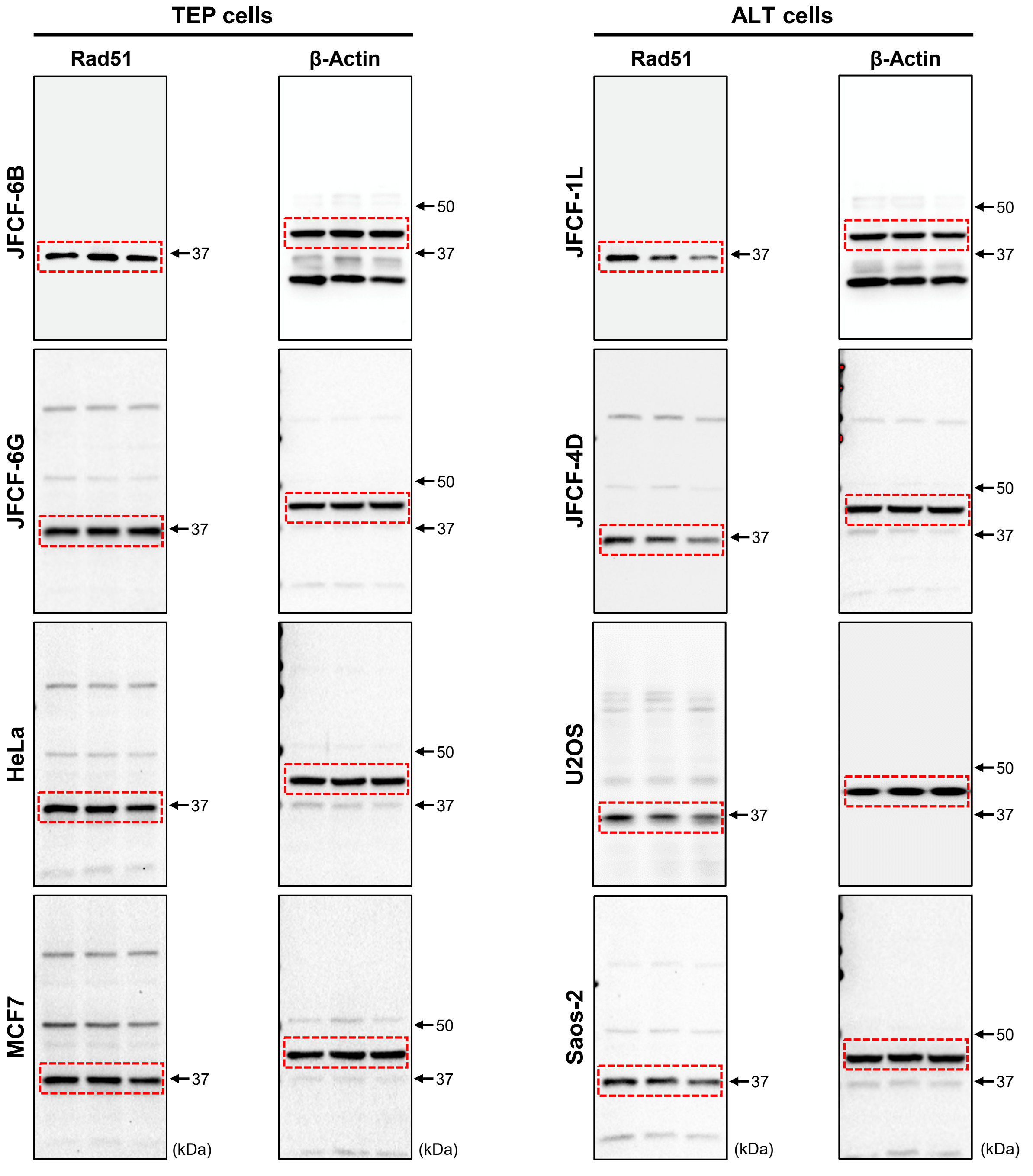
TEP cells
ALT cells
Rad51
β-Actin
Rad51
β-Actin
JFCF-6B
JFCF-6G
HeLa
MCF7
JFCF-1L
JFCF-4D
U2OS
Saos-2
50
37
(kDa)

Figure 5B

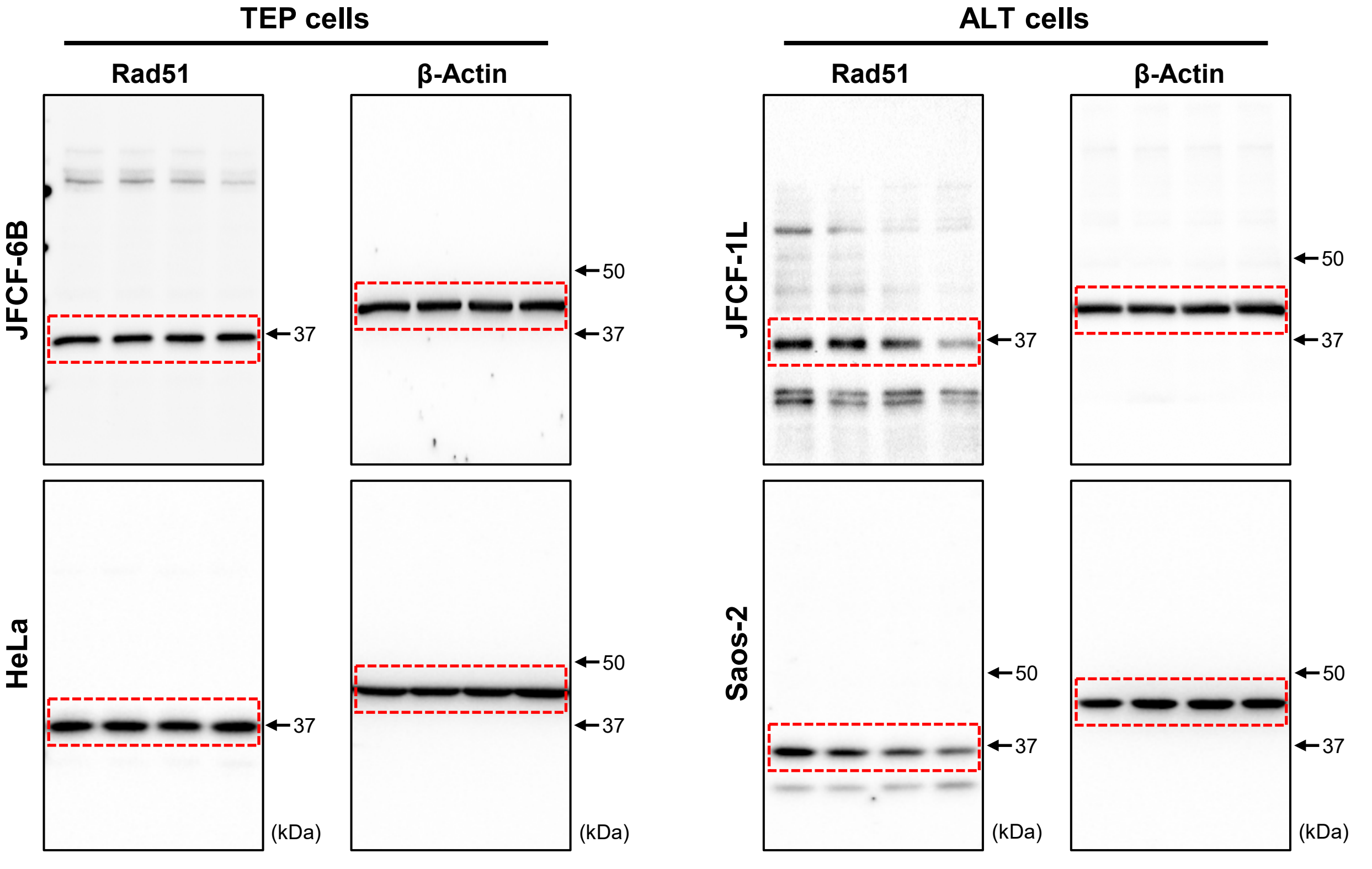

Figure 5C

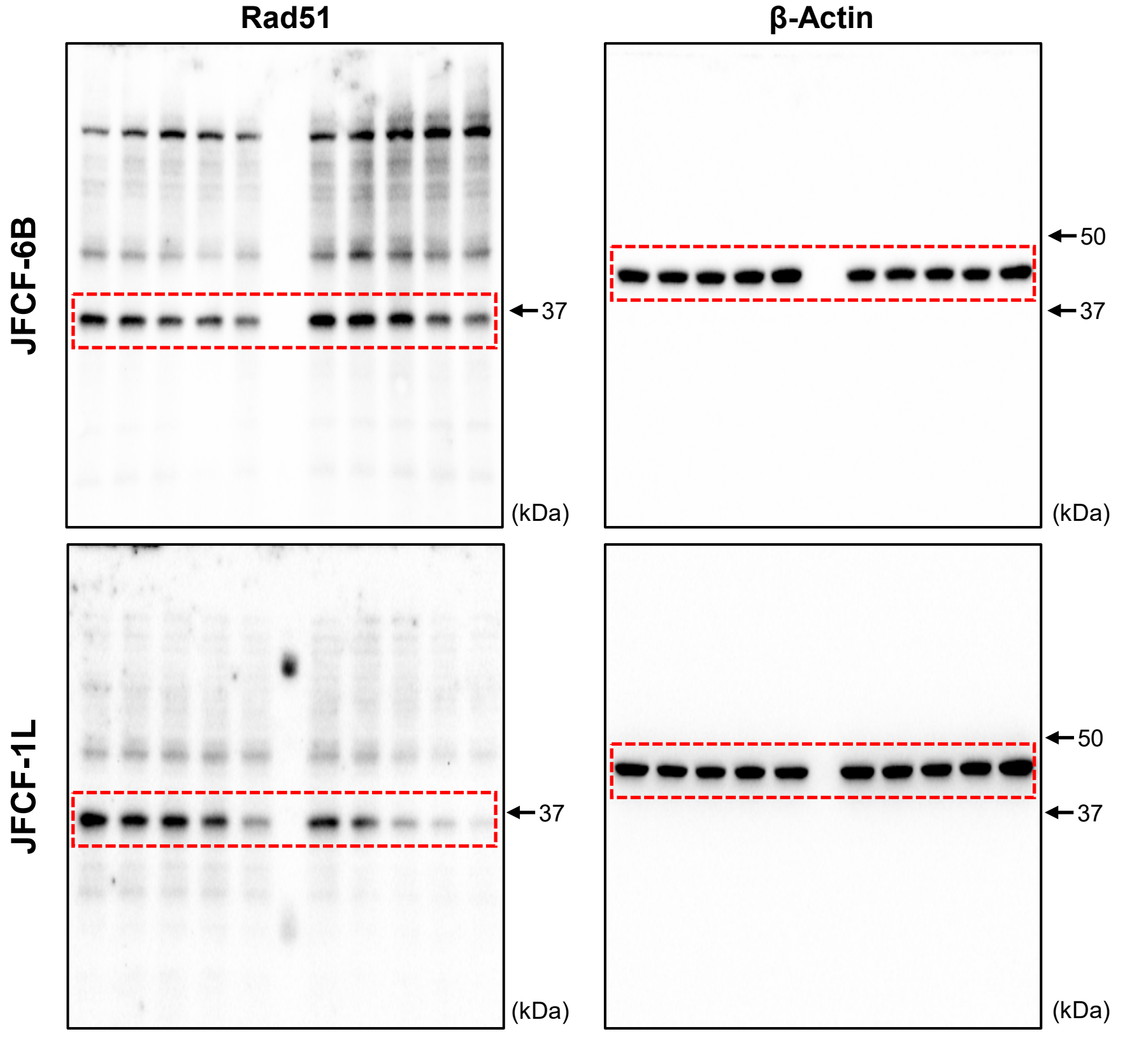

Figure 5E

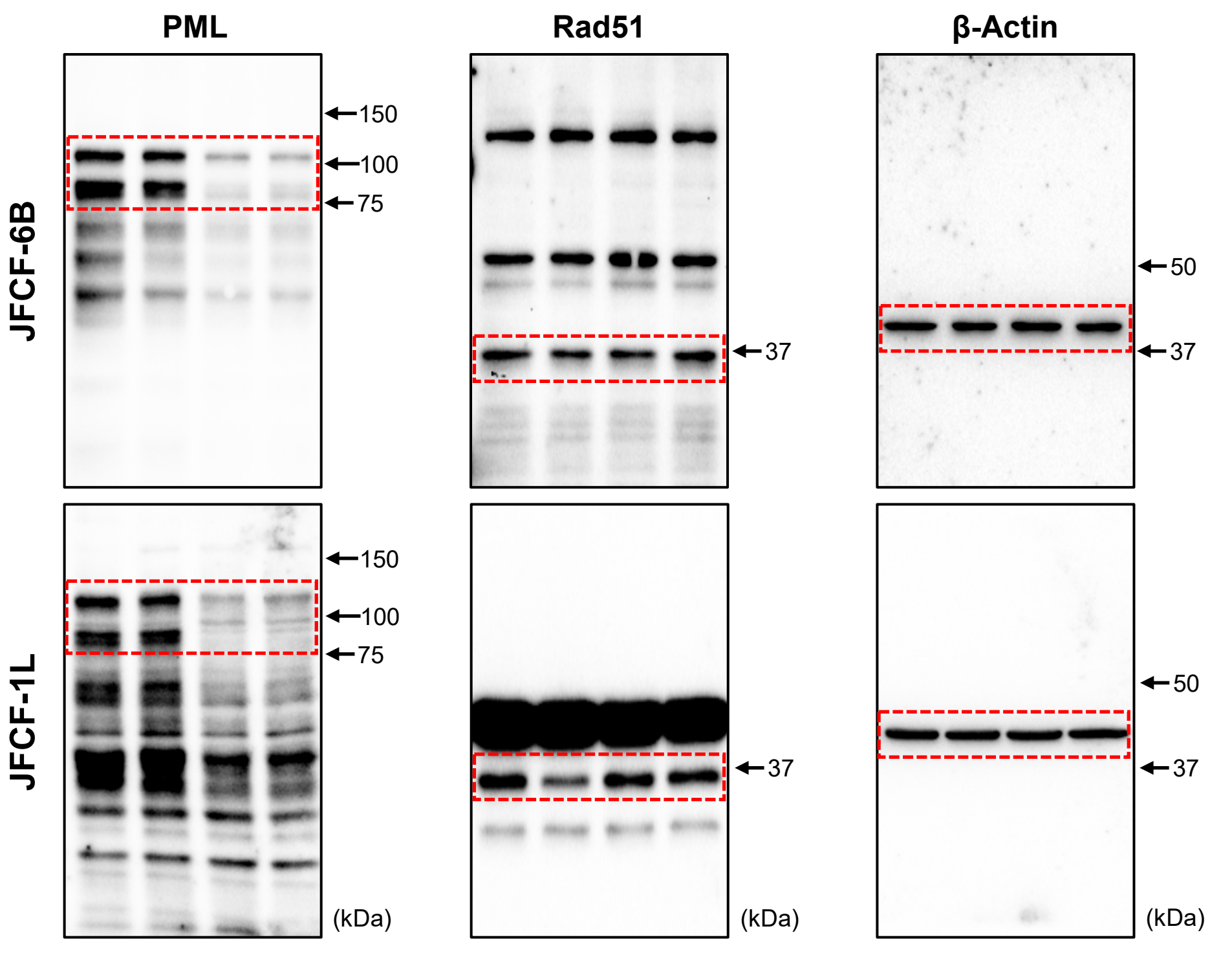

Figure 5F

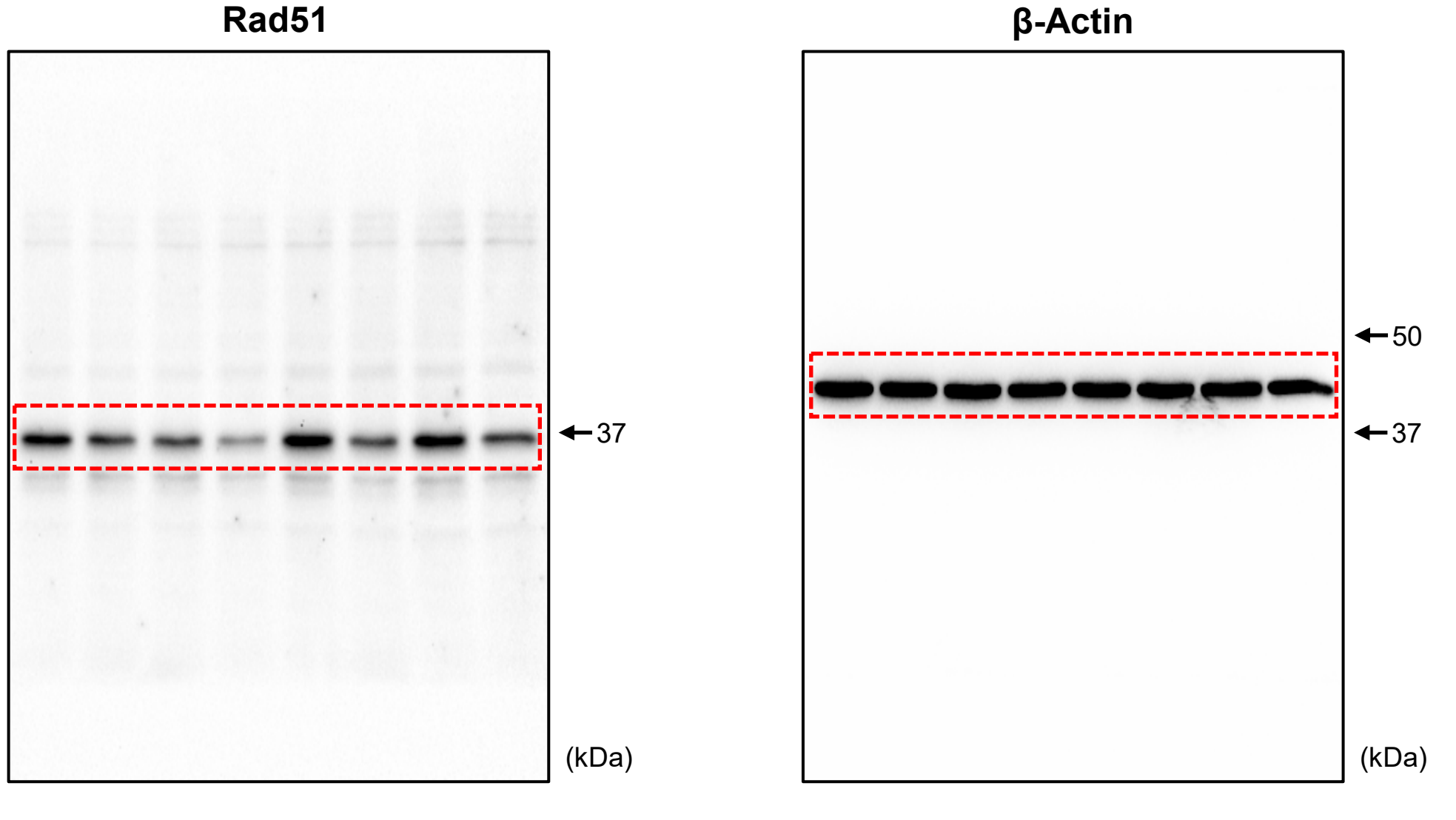

Figure S5D

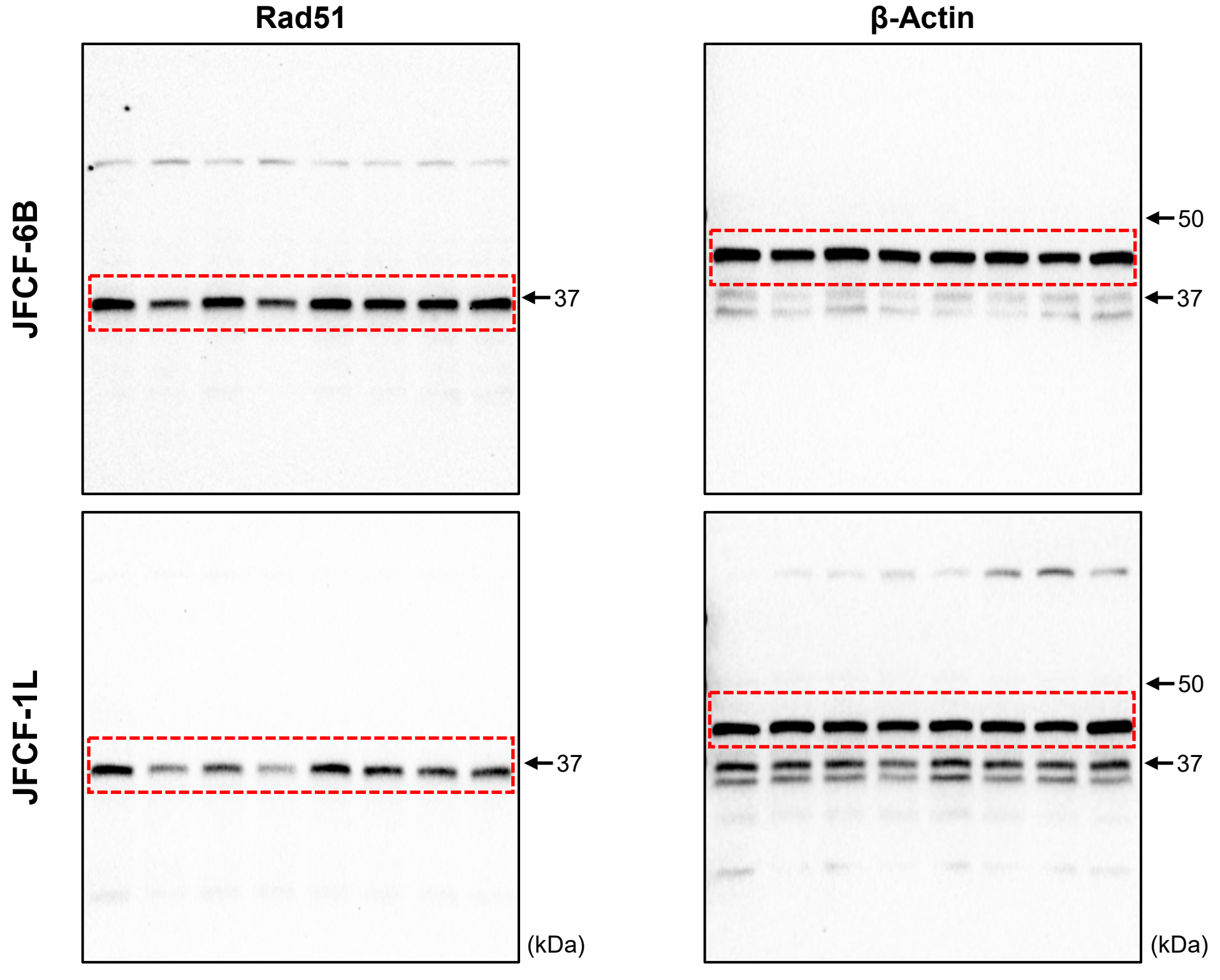

Figure 6C

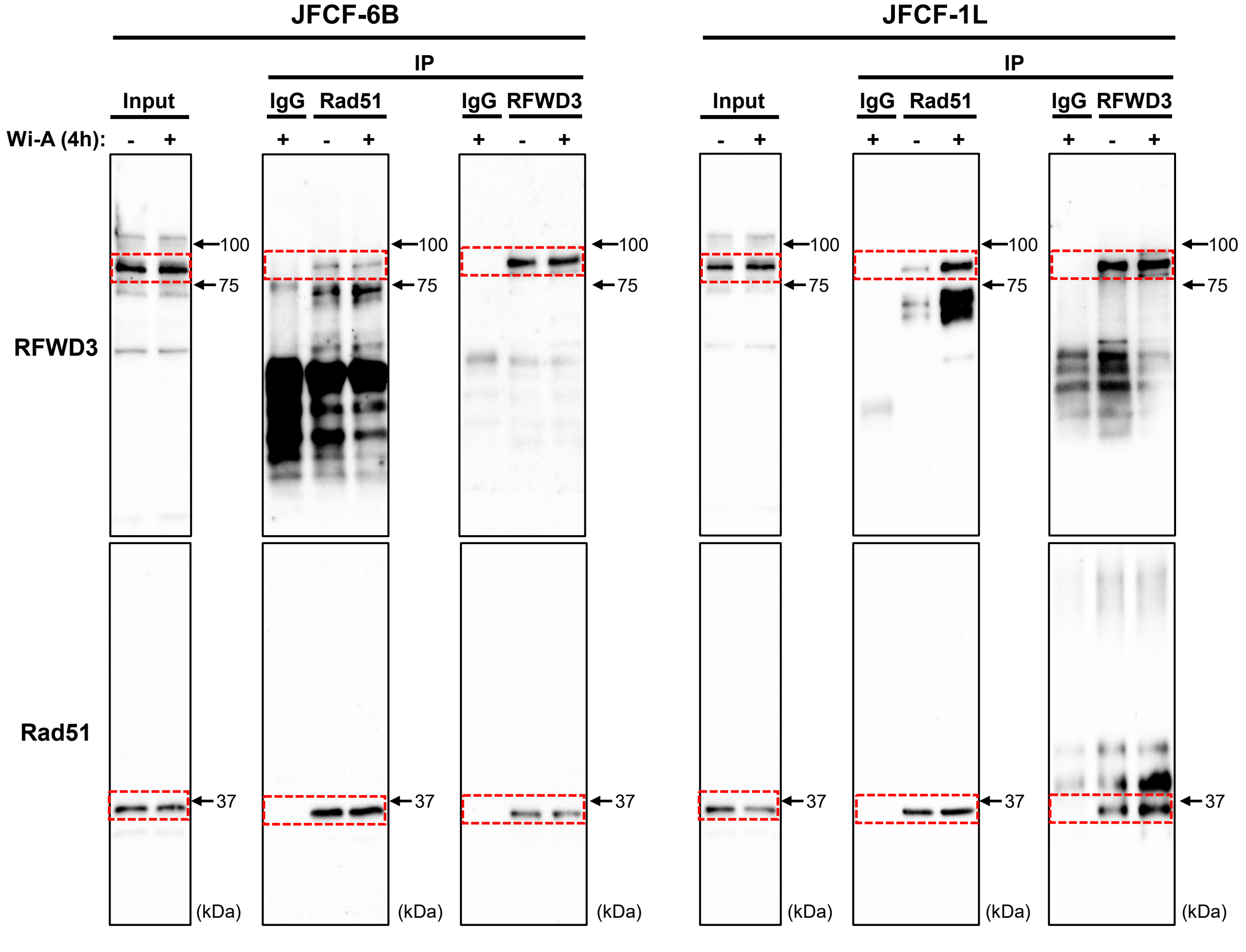

Figure 6F

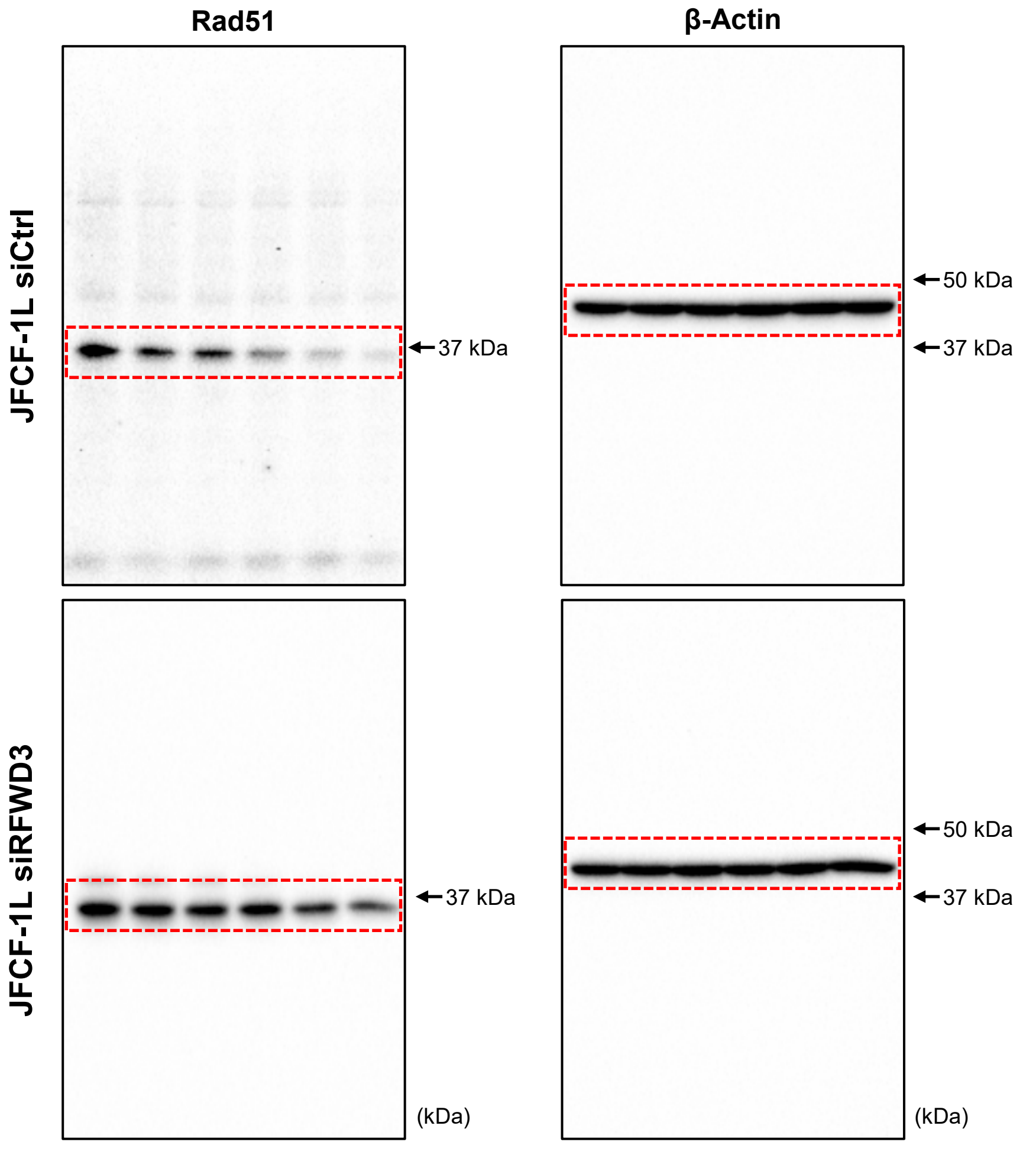

Figure S6

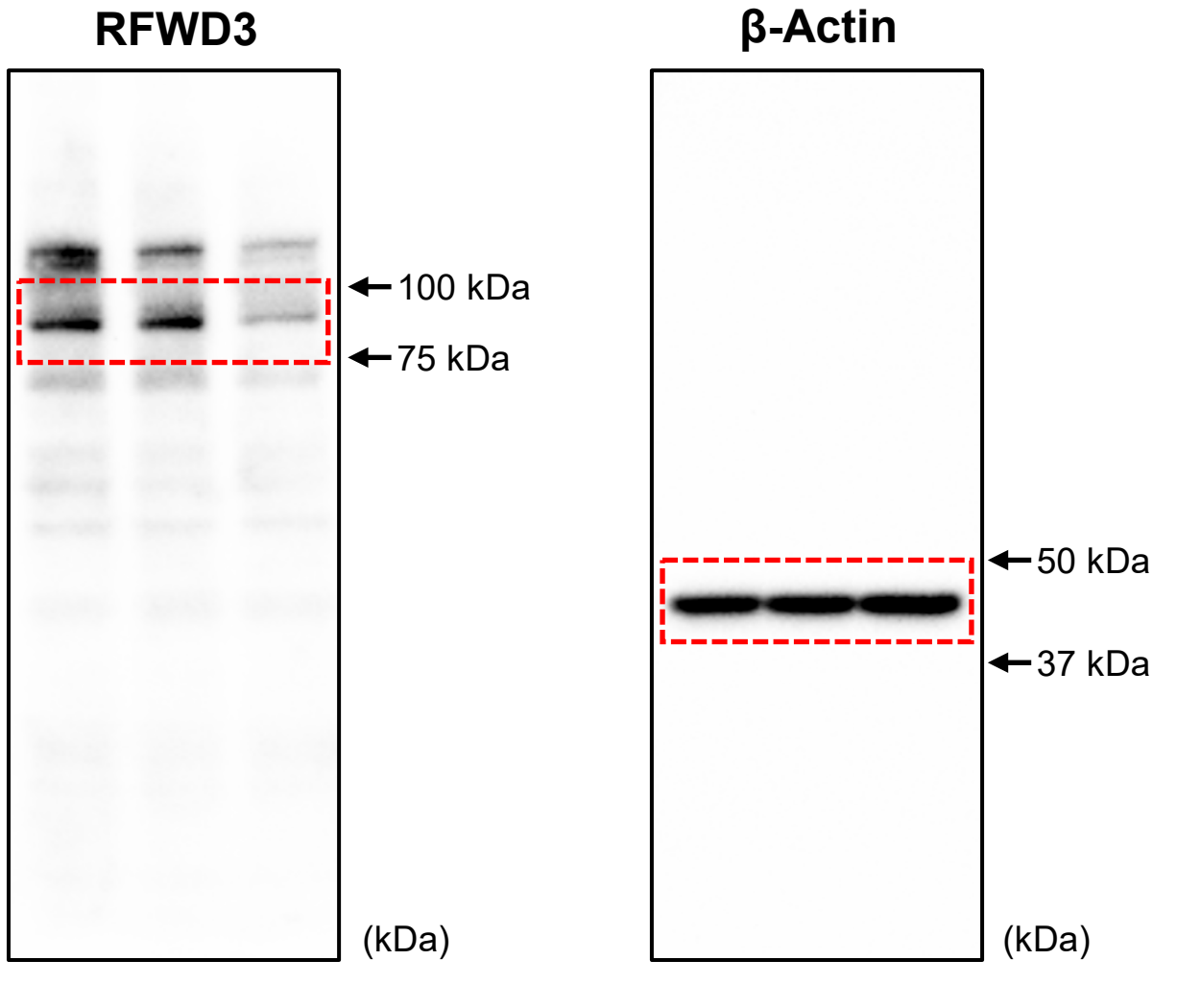

Figure S7A

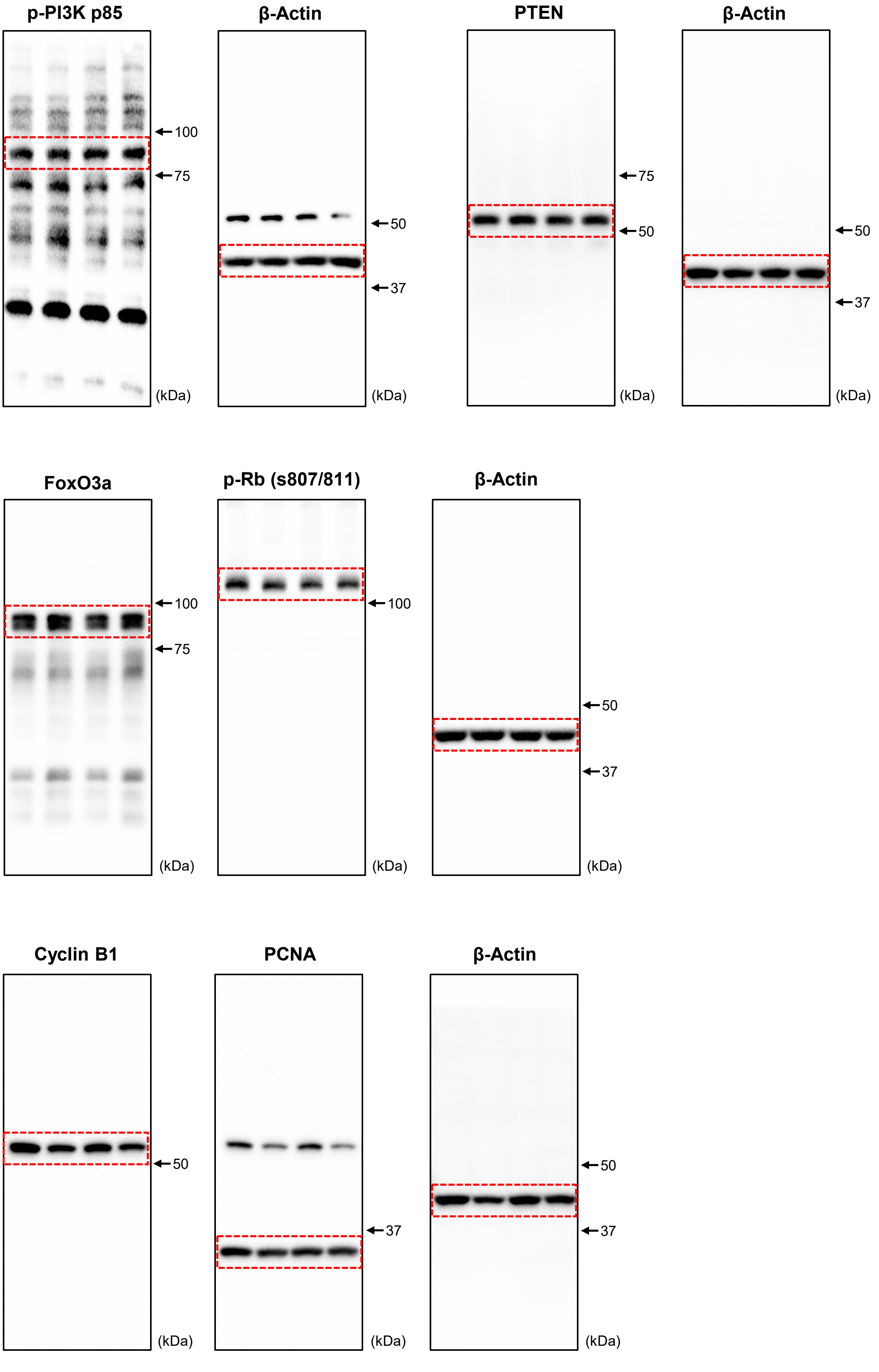

p-PI3K p85
β-Actin
PTEN
β-Actin
FoxO3a
p-Rb (s807/811)
β-Actin
Cyclin B1
PCNA
β-Actin
100
75
50
37
(kDa)

Figure S7B

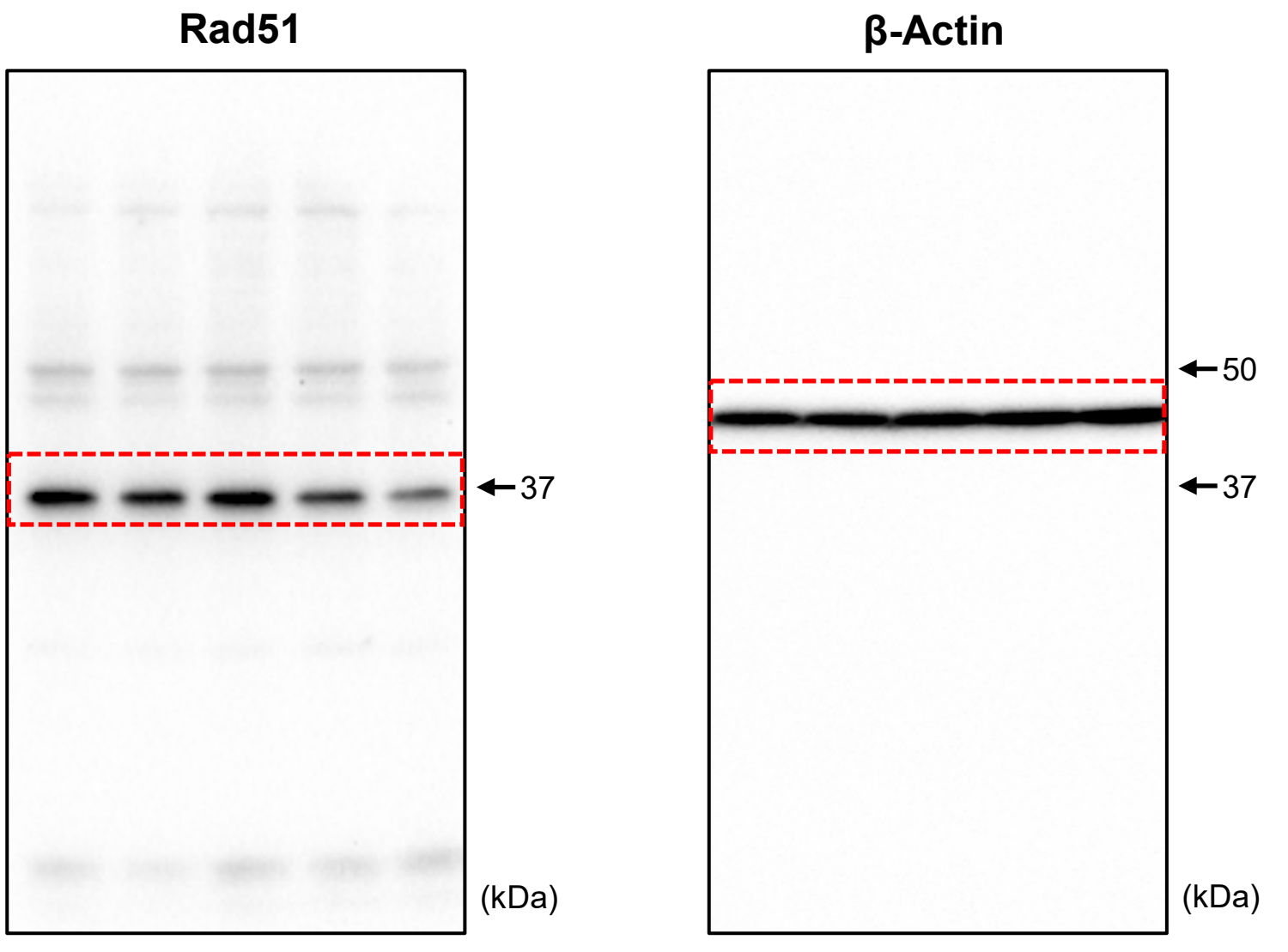

Figure S7C

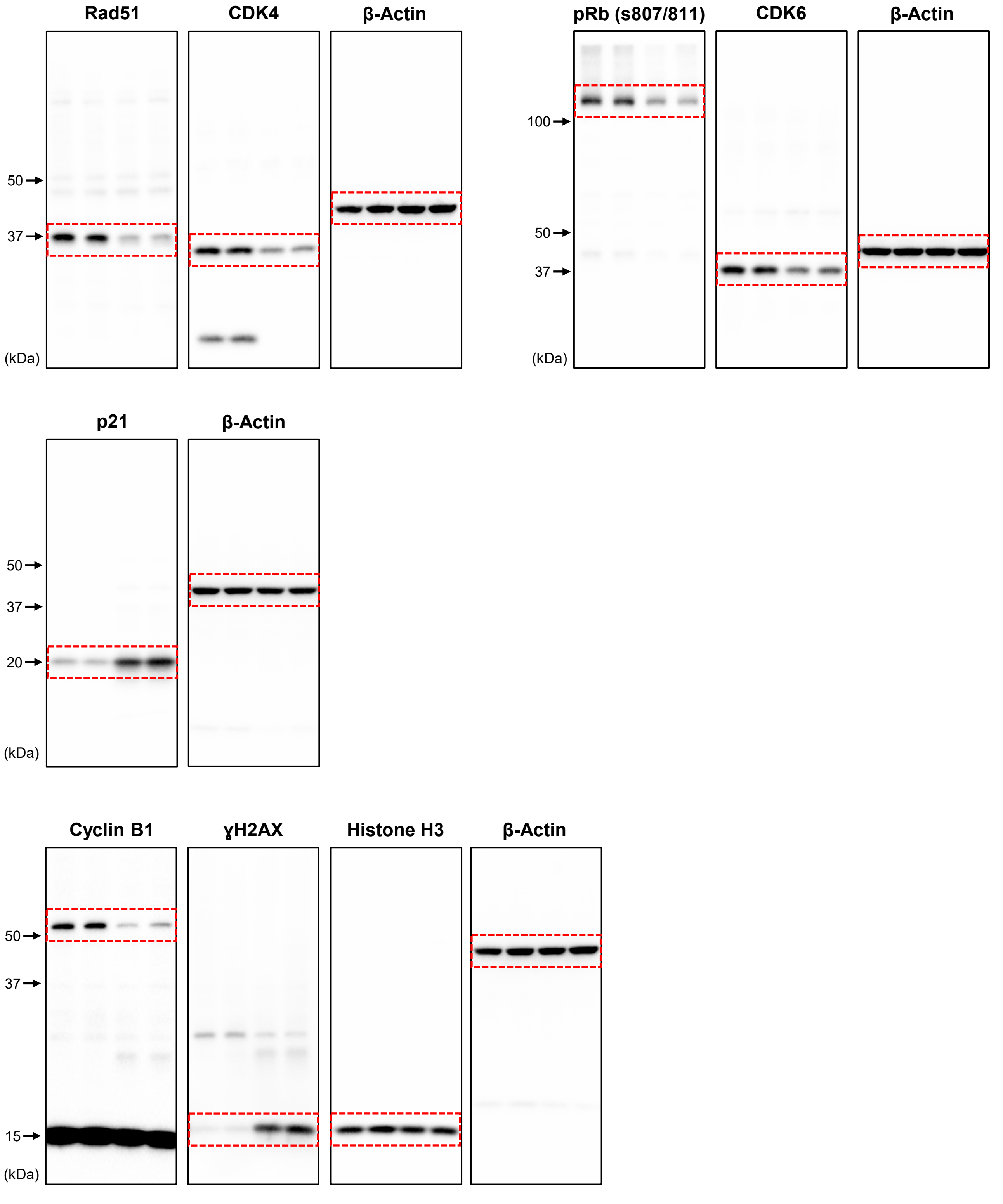
Rad51
CDK4
β-Actin
pRb (s807/811)
CDK6
β-Actin
50
37
(kDa)
100
50
37
(kDa)
p21
β-Actin
50
37
20
(kDa)
Cyclin B1
γH2AX
Histone H3
β-Actin
50
37
15
(kDa)
