# Supplementary material for: Selective killing of ALT-cancer cells by Withaferin-A involves destabilization of Rad51 and disruption of DNA repair
Source: Cell Death Dis. 2026 Jul 15;17(1):873. doi: 10.1038/s41419-026-09115-z (PMC13649652; doi:10.1038/s41419-026-09115-z)
Supplement: Supplementary file 2 [file 41419_2026_9115_MOESM1_ESM.docx]

**Supplementary information for**

**Selective killing of ALT-cancer cells by Withaferin-A involves destabilization of Rad51 and disruption of DNA repair**

Yue Yu^1*†^, Huayue Zhang^2†^, Jia Wang^2^, Jaspreet K Dhanjal^2^, Vidhi Malik^2,3^, Xiupeng Wang^4^, Sunil C Kaul^2^, Durai Sundar^3,5^, Renu Wadhwa^2*^

^1^Molecular Biosystems Research Institute, National Institute of Advanced Industrial Science and Technology (AIST), Tsukuba 305-8565, Japan

^2^AIST-INDIA DAILAB, Cellular and Molecular Biotechnology Research Institute, AIST, Tsukuba 305-8565, Japan

^3^DAILAB, Department of Biochemical Engineering & Biotechnology, Indian Institute of Technology (IIT) Delhi, New Delhi 110-016, India

^4^ Health and Medical Research Institute, AIST, Tsukuba 305-8565, Japan

^5^Institute of Bioinformatics and Applied Biotechnology (IBAB), Bengaluru 560-100, India

^†^These authors contributed equally to this work.

^*^To whom correspondence should be addressed:

Email: [yu-yue@aist.go.jp](mailto:yu-yue@aist.go.jp) (Y. Y.)

Email: [renu-wadhwa@aist.go.jp](mailto:renu-wadhwa@aist.go.jp) (R. W.)

**Table of contents:**

**Supplementary Figures 1 to 7**

**Supplementary Tables 1 to 3**

**Figure S1 (A)** Soft clustering analysis with Mfuzz. Genes in clusters 4 and 9 were selected for enrichment analysis because they showed marked Wi-A-induced expression changes in ALT (JFCF-1L) cells but remained unchanged in TEP (JFCF-6B) cells. Pathway enrichment analysis of genes in clusters 4 **(B)** and 9 **(C)** (KEGG database). PI3K-Akt signaling (known to be involved in carcinogenesis), containing 39 dysregulated genes, ranked among the top enriched pathways, indicating its strong contribution to the Wi-A-associated anti-cancer activity in ALT cells.

**Figure S2 (A)** Cell proliferation by colony forming assay in control and Wi-A-treated cells showing stronger response of ALT cells to Wi-A treatment (0.5 μM for 24 h). Number of crystal violet stained colonies was quantified on the right (mean ± s.d., n = 3 independent experiments, Student’s two-tailed t-test). **(B)** Cell cycle analysis showing increase in cell population at G2/M arrest in ALT cells by Wi-A treatment (0.5 μM for 24 h). TEP cells do not show a similar increase with the same treatment. Quantitation for cells in G2/M phase is shown on the right (mean ± s.d., n = 3 independent experiments, Student’s two-tailed t-test). **(C-E)** Relative protein expression levels quantified from Western blot analyses. **(C)** Quantitation for Figure 2D. **(D)** Quantitation for Figure 2F. **(E)** Quantitation for Figure 2E. Statistical comparisons were performed against the corresponding controls using a two-tailed Student’s t-test (mean ± s.d., n = 3 independent experiments). γH2AX was normalized to Histone H3.

**Figure S3 (A)** Schematic of the experimental design. Cells were treated with Wi-A (0.5 μM) for 24 h, followed by exposure to 2 Gy IR. After recovery in drug-free medium for 1 or 24 h, cells were harvested for Western blot analysis. **(B and C)** Western blot of γH2AX showing Wi-A does not affect the recovery of TEP cells (JFCF-6B) from IR-induced DNA damage but impairs recovery in ALT cells (JFCF-1L). Quantitation was normalized to Histone H3 and compared using a two-tailed Student’s t-test (mean ± s.d., n = 3 independent experiments).

**Figure S4 (A)** Superimposition of Rad51-BRCA2 complex (Grey-blue) with that of Rad51-BRCA2-Wi-A complex (Yellow-orange-red). **(B)** ATP levels in TEP and ALT cells following Wi-A treatment (0.5 μM, 24 h) showing no significant change. Results were quantified from three independent experiments (mean ± s.d.). **(C)** Western blotting of γH2AX showing that 4 h Wi-A treatment (0.5 μM) induced DNA damage, which persisted in ALT cells but resolved in TEP cells with prolonged treatment to 24 h. Quantitation from three independent experiments is shown on the right (mean ± s.d., normalized to Histone H3, one-way ANOVA test).

**Figure S5 (A and B)** Relative Rad51 expression levels quantified from Figures 5A and 5B. Statistical significance was determined using one-way ANOVA test (mean ± s.d., n = 3 independent experiments). **(C)** RT-qPCR analysis showing no significant change in Rad51 mRNA levels in TEP (JFCF-6B) and ALT (JFCF-1L) cells following 4 h Wi-A treatment (0.5 μM). Data from three independent experiments was graphed as the mean ± s.d.. **(D)** Wi-A promotes Rad51 destabilization in ALT cells via proteasomal degradation. Cells were treated with 5 μM MG132, 0.5 μM Wi-A, 10 μg/mL CHX, or their combinations for 4 h, and analyzed by western blotting. Quantitation of Rad51 protein levels is shown on the right (mean ± s.d., n = 3 independent experiments). **(E and F)** Immunofluorescence analysis showing that Rad51 is recruited to damaged telomeres induced by 4h Wi-A treatment (0.5 μM) in both TEP and ALT cells. Telomeres were stained with an antibody against TRF1 and TRF2. Quantification of at least 200 randomly selected nuclei per treatment is shown on the right (one-way ANOVA test). **(G and H)** Relative protein levels quantified from Figures 5E and 5F. Statistical significance was determined using one-way ANOVA test for panel G and two-way ANOVA for panel H (mean ± s.d., n = 3 independent experiments).

**Figure S6** Western blotting of RFWD3 confirming its siRNA knockdown efficiency. JFCF-1L cells were mock-treated or transfected with control or RFWD3 siRNAs and harvested 24 hours post-transfection. Quantitation is shown on the right (mean ± s.d., n = 3 independent experiments, one-way ANOVA test).

**(B)** Relative changes of Rad51 over time quantified from Figure 6F (mean ± s.d., n = 3 independent experiments). The ratio of Rad51 to β-actin band intensity was normalized to the starting point of the chase.

**Figure S7 (A)** Western blot analysis of PI3K and proliferation signaling proteins showing no significant differences between isogenic TEP and ALT cells. Quantification is shown on the right (mean ± s.d., n = 3 independent experiments). **(B)** Growth curve of JFCF-1L cells treated with Wi-A (0.5 μM, 8 h). Rad51 levels were assessed by Western blot at indicated time points (below). Quantification from three independent experiments is shown (mean ± s.d., one-way ANOVA test). **(C)** Western blot analysis of JFCF-1L cells showing decreased expression of proliferation markers and increased γH2AX following Rad51 knockdown. Cells were harvested 48 h after siRNA transfection. Quantification is shown on the right (mean ± s.d., n = 3 independent experiments, one-way ANOVA test). **(D)** Colony formation assay of JFCF-1L cells showing increased sensitivity to DNA damage following Rad51 inhibition. Cells were treated with RI-1 (5 μM) or transfected with siRNA for 24 h, followed by exposure to cisplatin (0.2 μM, 4 h) or ionizing radiation (2 Gy), and then cultured in drug-free medium for 12 days. Colonies were quantified and are shown on the right (mean ± s.d., n = 3 independent experiments, two-tailed Student’s t-test).

**Table S1** List of interactions formed by ATP and Wi-A at Rad51 protomer-protomer interface and their binding energy at ATPase domain. Common interacting residues are highlighted in bold.

| **Properties** | **Rad51-ATP-Rad51**  **Complex** | | **Rad51-WiA-Rad51**  **Complex** | |
| --- | --- | --- | --- | --- |
| MM-GBSA (ligand) binding energy (Kcal/mol) | -35.43 | | -41.02 | |
|  |  |  |  |  |
| Rad51-Rad51 binding energy (Kcal/mol) | -15.8 | | -17.7 | |
|  |  |  |  |  |
| Ligand interactions with protein | Chain A | Chain B | Chain A | Chain B |
| Hydrogen Bonds | **Cys319** | Arg130 | - | - |
|  |  | Lys133 |  |  |
|  |  | Thr134 |  |  |
|  |  | Gln135 |  |  |
|  |  | Gln268 |  |  |
|  |  | Arg310 |  |  |
| Hydrophobic Interactions | Ser317 | Phe129 | Lys111 | **Arg170** |
|  | **Leu320** | Gly132 | Gln114 | **Ala331** |
|  | **Pro321** | **Arg170** | **Cys319** |  |
|  | Glu322 | Ile329 | **Leu320** |  |
|  |  | Asn330 | **Pro321** |  |
|  |  | **Ala331** |  |  |

**Table S2 Antibodies used in this study**

| **Antibody** | **Type** | **Source** | **Catalog No.** | **Application** |
| --- | --- | --- | --- | --- |
| PI3 Kinase p85 | Rabbit monoclonal | Cell Signaling | 4257 | WB |
| PTEN | Mouse monoclonal | Santa Cruz | sc-56205 | WB |
| Phospho-PI3 Kinase p85 (Tyr458)/p55 (Tyr199) | Rabbit monoclonal | Cell Signaling | 4228 | WB |
| Akt | Rabbit monoclonal | Cell Signaling | 4691 | WB |
| Phospho-Akt (Ser473) | Rabbit monoclonal | Cell Signaling | 4060 | WB |
| FoxO3a | Rabbit monoclonal | Cell Signaling | 2497 | WB, IF |
| Cyclin D1 | Mouse monoclonal | Santa Cruz | sc-56302 | WB |
| Cyclin B1 | Mouse monoclonal | Santa Cruz | sc-245 | WB |
| Cdk1 | Mouse monoclonal | BD Biosciences | 610037 | WB |
| Cdk4 | Rabbit polyclonal | Santa Cruz | sc-260 | WB |
| Cdk6 | Rabbit polyclonal | Santa Cruz | sc-177 | WB |
| Phospho-Rb (Ser780) | Rabbit polyclonal | Cell Signaling | 9307 | WB |
| Phospho-Rb (Ser807/811) | Rabbit polyclonal | Cell Signaling | 9308 | WB |
| PCNA | Mouse monoclonal | Abcam | ab29 | WB |
| p53 | Rabbit monoclonal | Cell Signaling | 2527 | WB |
| Phospho-p53 (Ser15) | Rabbit polyclonal | Cell Signaling | 9284 | WB |
| p21 Waf1/Cip1 | Rabbit monoclonal | Cell Signaling | 2947 | WB |
| CARF | Rabbit polyclonal | Homemade | FL-A10 | WB |
| 53BP1 | Rabbit polyclonal | Abcam | ab21083 | IF |
| ɣH2AX (Ser139) | Rabbit monoclonal | Cell Signaling | 9718 | WB, IF |
| ɣH2AX (Ser139) | Mouse monoclonal | Abcam | ab22551 | WB |
| TRF2 | Mouse monoclonal | Santa Cruz | sc-52968 | IF |
| TRF2 + TRF1 | Mouse monoclonal | Abcam | ab10579 | IF |
| Rad51 | Mouse monoclonal | Santa Cruz | sc-53428 | WB |
| Rad51 | Rabbit monoclonal | Abcam | ab133534 | WB, IF, IP |
| RPA32 | Rat monoclonal | Cell Signaling | 2208 | WB |
| RPA70 | Rabbit polyclonal | Cell Signaling | 2267 | WB |
| PML | Mouse monoclonal | Santa Cruz | sc-966 | WB, IF |
| PML | Goat polyclonal | Santa Cruz | sc-9862 | WB |
| RFWD3 | Rabbit polyclonal | Thermo Fisher | PA5-83903 | IF |
| RFWD3 | Rabbit polyclonal | Abcam | ab138030 | WB |
| RFWD3 | Rabbit polyclonal | Abcam | ab99306 | IP |
| Histone H3 | Mouse monoclonal | Santa Cruz | sc-517576 | WB |
| Fibrillarin | Mouse monoclonal | Santa Cruz | sc-166001 | WB |
| α-tubulin | Mouse monoclonal | Sigma | T5168 | WB |
| β-actin, HRP | Mouse monoclonal | Biolegend | 643807 | WB |
| Anti-rabbit IgG, HRP | Goat polyclonal | Thermo Fisher | 31460 | WB |
| Anti-mouse IgG, HRP | Goat polyclonal | Thermo Fisher | 31430 | WB |
| Anti-goat IgG, HRP | Rabbit polyclonal | Thermo Fisher | 31402 | WB |
| Anti-rat IgG, HRP | Goat polyclonal | Santa Cruz | sc-2006 | WB |
| Anti-Rabbit IgG, Alexa Fluor 488 | Goat polyclonal | Thermo Fisher | A-11034 | IF |
| Anti-Rabbit IgG, Alexa Fluor 594 | Donkey polyclonal | Thermo Fisher | A-21207 | IF |
| Anti-Mouse IgG, Alexa Fluor 488 | Goat polyclonal | Thermo Fisher | A-32723 | IF |
| Anti-Mouse IgG, Alexa Fluor 594 | Goat polyclonal | Thermo Fisher | A-11032 | IF |
| Normal Rabbit IgG | Rabbit polyclonal | Cell Signaling | 2729 | IP |

**Table S3 Primers used in this study**

| **Gene** | **Forward primer (5’→3’)** | **Reverse primer (5’→3’)** |
| --- | --- | --- |
| Rad51 | TGGCCCACAACCCATTTCAC | TCAATGTACATGGCCTTTCCTTCAC |
| CCND2 | TTTGCCATGTACCCACCGTC | AGGGCATCACAAGTGAGCG |
| COL1A2 | TCAAACTGGCTGCCAGCAT | CAAGAAACACGTCTGGCTAGG |
| COL4A2 | TGTCTCCATCCCACACTGCC | CCTCTAGACAGCTGCCCGGT |
| COL5A1 | CGCTCTCCCGTCTTCCTC | CACCCTCAAACACCTCCTCA |
| SGK2 | TGTGACAGGACCTGCTGACTTGAA | AGATGTCATCATCCTCTGGCGCAT |
| SPP1 | GAGGGCTTGGTTGTCAGC | CAATTCTCATGGTAGTGAGTTTTCC |
| MYB | GGGAACAGATGGGCAGAAATCG | GCTGGCTTTTGAAGACTCCTGC |
| 1L2Rβ | ACTCGAGAGCCAACATCTCC | TCCGAGGATCAGGTTGCAG |
| 18S | CAGGGTTCGATTCCGTAGAG | CCTCCAGTGGATCCTCGTTA |
